# Leaf out time correlates with wood anatomy across large geographic scales and within local communities

**DOI:** 10.1111/nph.18041

**Published:** 2022-03-12

**Authors:** Jessica A. Savage, Thomas Kiecker, Natalie McMann, Daniel Park, Matthew Rothendler, Kennedy Mosher

**Affiliations:** ^1^ Department of Biology University of Minnesota Duluth MN 55812 USA; ^2^ Department of Biological Sciences Purdue University West Lafayette IN 47907 USA; ^3^ Department of Biology Boston University Boston MA 02215 USA

**Keywords:** freezing‐induced embolism, leaf out, leaf phenology, vascular phenology, vessel, wood porosity, xylem

## Abstract

- There is a long‐standing idea that the timing of leaf production in seasonally cold climates is linked to xylem anatomy, specifically vessel diameter because of the hydraulic requirements of expanding leaves.
- We tested for a relationship between the timing of leaf out and vessel diameter in 220 plants in three common gardens accounting for species’ phylogenetic relationships. We investigated how vessel diameter related to wood porosity, plant height and leaf length. We also used dye perfusion tests to determine whether plants relied on xylem produced during the previous growing season at the time of leaf out.
- In all three gardens, there was later leaf out in species with wider vessels. Ring‐porous species had the widest vessels, exhibited latest leaf out and relied less on xylem made during the previous growing season than diffuse‐porous species. Wood anatomy and leaf phenology did not exhibit a phylogenetic signal.
- The timing of leaf out is correlated with wood anatomy across species regardless of species’ geographic origin and phylogenetic relationships. This correlation could be a result of developmental and physiological links between leaves and wood or tied to a larger safety efficiency trade‐off.

## Abstract 2

See also the Commentary on this article by Olson, **235**: 815–820.

## Introduction

Spring phenology is advancing across the globe in response to climate change especially in nontropical climate zones. A major component of this shift is driven by early leaf out in deciduous species (Menzel & Fabian, 1999; Cleland *et al*., 2007; Morin *et al*., 2009; Polgar & Primack, 2011). Early leaf out can be beneficial because ‘tracking’ the climate as it warms (i.e. adjusting phenology to new climatic conditions) can lead to greater growth and/or reproduction in some species (Cleland *et al*., 2012). However, early leaf out can also be problematic if it occurs during a period of warming followed by a hard frost, known as a false spring (Inouye, 2008; Hufkens *et al*., 2012). In the end, what matters is the ‘match’ between phenology and the environment, and this ‘match’ is influenced by species‐specific differences in anatomy and physiology (Cooke *et al*., 2012; Panchen *et al*., 2014; Flynn & Wolkovich, 2018). Recent meta‐analyses suggest that one characteristic important in explaining differences in leaf phenology among species, specifically the timing of leaf out, is wood anatomy (Panchen *et al*., 2014; Fahey, 2016).

Wood is composed of the xylem, which is the part of the vascular system responsible for transporting water, and its structure has important implication for plant stress tolerance and growth (Hacke & Sperry, 2001; Chave *et al*., 2009; Venturas *et al*., 2017). Because leaves are hydrated by water transported from the roots through the xylem, stem xylem conductance is related to a variety of leaf traits from leaf primordia number (Cochard *et al*., 2005) to leaf water loss (Saliendra *et al*., 1995; Hubbard *et al*., 2001; Tyree & Zimmerman, 2002). In a critical paper on leaf phenology, Lechowicz (1984) proposed that one of the reasons that plants within a forest exhibit differences in their timing of leaf out is because of variation in their wood anatomy that can impact xylem transport capacity in the spring. When sap freezes, dissolved gases form bubbles that can expand and embolize transport conduits (Sperry & Sullivan, 1992; Sperry & Pockman, 1993; Sevanto *et al*., 2012). The likelihood of embolism formation is closely tied to wood anatomy because larger bubbles have a greater chance of expanding, and bubble size can increase with conduit diameter (Yang & Tyree, 1992; Davis *et al*., 1999). As a result, plants with narrow conduits (vessels and/or tracheids) that experience limited embolism will have a high xylem conductance and should be able to support a new canopy early in the spring. Meanwhile, plants with wide conduits and reduced xylem conductance must wait until the formation of new conduits (xylogenesis) to leaf out. This framework has been used to explain why there is variation in leaf phenology among co‐occurring species (Wang *et al*., 1992), why plants with narrow conduits appear more plastic in their leaf phenology when compared to species with wider conduits (Fahey, 2016), and why plants from colder and warmer climates exhibit differences in their vessel width even when they have comparable heights (M. Olson *et al*., 2020).

In the original paper, Lechowicz (1984) presented preliminary data on the potential relationship between leaf phenology and wood anatomy and later collaborated on a study examining the relationship between leaf phenology and xylem embolism in trees (Wang *et al*., 1992). Despite the importance of these original studies, they relied on wood anatomy/physiology measured on different plants than those monitored for phenology. We now know this type of comparison can be problematic because xylem anatomy, specifically conduit width, is highly influenced by plant height, sampling location within the plant, and growth conditions (Choat *et al*., 2007; Prislan *et al*., 2013; M. E. Olson *et al*., 2020). There is also evidence that because water potential impacts bubble expansion (Pittermann & Sperry, 2006), embolism formation could be influenced by tree height and environmental conditions (Mayr *et al*., 2003; Charrier *et al*., 2017). Recently, studies have found conflicting results about whether wood anatomy relates to leaf phenology (Yin *et al*., 2016; Osada, 2017) that may be a result of differences in sampling design.

The idea of a link between xylem anatomy and leaf phenology has been perpetuated over the years because it is appealing from a functional perspective and ties into larger discussions of leaf–stem relationships and how they mediate phenology across taxa and climate zones (Méndez‐Alonzo *et al*., 2012; M. E. Olson *et al*., 2020). If a relationship between wood anatomy and leaf phenology proves robust across taxa, it would allow wood anatomy to serve as a proxy for leaf phenology in larger studies. However, strictly from a hydraulic perspective, there are many reasons to expect this relationship to be weak across taxa. One reason is that species should exhibit differences in how much functional xylem they require at leaf out depending on their leaf water use, leaf area, and the coordination between new wood development and leaf expansion (see discussion in Savage & Chuine (2021)). If some species require significantly less conductive xylem tissue to support their leaves than other species, then leaf out time might not correlate well with wood anatomy across species. Another key assumption of the proposed relationship between leaf phenology and conduit diameter is that average conduit diameter predicts xylem conductance in the spring. This assumption can break down if species exhibit refilling of embolized conduits (discussed in Wang *et al*. (1992)). The situation is further complicated by heterogeneity in conduit size within a piece of wood (discussed in Yin *et al*. (2016)) and the scaling of conduits within a tree (Mencuccini, 2002; Olson & Rosell, 2013; Lechthaler, 2020), because wider conduits have a larger impact on hydraulic conductance and vulnerability to freezing‐induced embolism than narrower conduits.

Here, we directly tested the relationship between leaf out time and vessel diameter in 220 woody flowering plants (angiosperms) that were grown in three common gardens. Our aim was to test whether this relationship exits and determine if wood anatomy can adequately predict leaf phenology. To account for plasticity and intraspecific variation, we observed phenology on the same plants that were used for anatomical analyses. We also investigated how phylogeny, species geographic origin, plant height and leaf length are related to both wood anatomy and leaf phenology. Overall, this study allows us to examine whether there might be developmental and physiological links that exist between stem xylem and leaf phenology.

## Materials and Methods

### Sampling and study sites

**Fig. 1:**
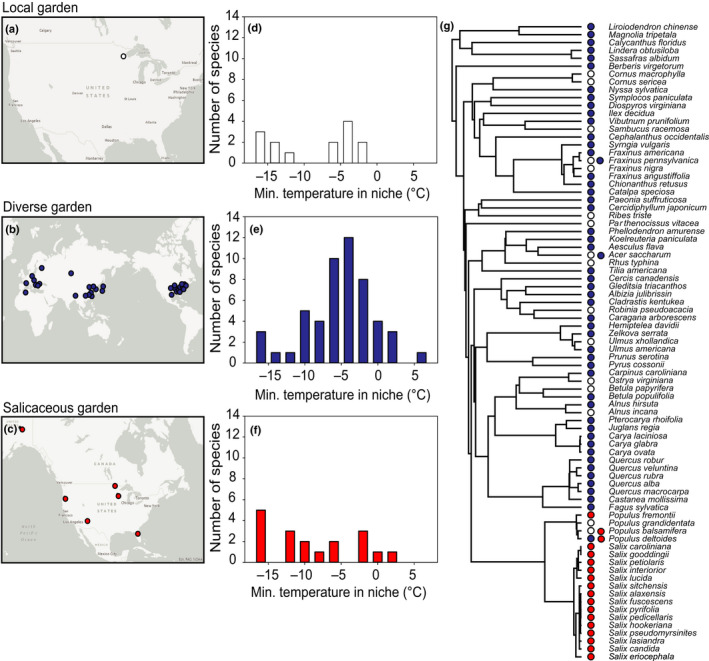
The (a–c) geographic origins, (d–f) climatic niches and (g) phylogenetic spread of species in the three gardens. Gardens are differentiated by colors: white, local garden; blue, diverse garden; red, salicaceous garden. The approximate origin of the plant material in each garden is noted in (a–c). When exact location data was not available, the origin was marked as the center of the species range. The maps were created using a Light gray canvas basemap (Esri, 2021). Histograms of the minimum temperature in the most suitable habitat for the species based on the Maxent model described in the Methods section are shown in (d–f). Phylogeny is based on the time‐calibrated phylogeny of seed plants estimated in Smith & Brown (2018).

To examine the potential relationship between spring leaf phenology and woody anatomy in the context of phylogenetic relationships and climate of origin, we studied plants from three common gardens (Fig. 1). Initially, we sampled 14 species that are common in mixed, temperate forests and all co‐occur in Bagley Nature Area in Duluth, MN, USA (46.8241°N, 92.0868°W). We referred to this as our local garden. At this site, we sampled four individuals per species (total plants = 56, Supporting Information Table S1). Species were selected to maximize the spread of leaf out times with two to three species selected each week in the early spring in 2019. Species exhibited a diversity of growth forms and wood porosities. Our second garden was more phylogenetically diverse and plants came from different geographic origins (hereafter referred to as the diverse garden). It consisted of one individual from each of 55 species growing at the Arnold Arboretum of Harvard University in Boston, MA, USA (42.3074°N, 71.1208°W), which were sampled in 2017 (Table S2). These species were collected and/or cultivated from around the world and varied in growth form, average size, and wood anatomy/porosity. The final garden was phylogenetically limited and consisted of 18 species from the family Salicaceae, hereafter referred to as the salicaceous garden (Table S3). Plants were collected from sites across North America, propagated by cutting, and grown under common conditions in Franklinville, NY, USA (42.3369°N, 78.4579°W) in 2009. This garden contained a mixture of trees, shrubs and sub‐shrubs, but there was less diversity in wood anatomy compared to the other two gardens. Five to seven individuals per species were studied (total plants = 109). Plants were initially propagated in a glasshouse and then kept outside over the winter and into the following spring (Savage & Cavender‐Bares, 2013).

### Phenology, height, and weather

In each common garden, we monitored leaf phenology every 2–7 d in the spring and recorded leaf out, which we defined as when at least three branches or 50% of the plant (whichever came first) had leaves unfolding with the final leaf shape at least partially visible. Any plant that experienced significant dieback was noted. We also measured the height of all the plants. Leaf out dates were reported as day of the year and degree days, cumulative thermal sum of the degrees above 0°C each day starting on January 1 (Heide, 1993). Weather data from the Arnold Arboretum and Franklinville, NY, USA were collected onsite. In Duluth, data was collected from the Chester Park station (KMNDULT7) and the Hunter’s Park station (KMNDULUT86). We used the National Oceanic and Atmospheric Administration (NOAA) US Climate Normals (2006–2020) data to determine when the probability of freezing was below 10% at each site (www.ncei.noaa.gov).

### Wood analysis

For the diverse garden and the local garden, 3‐yr‐old branches were sampled during the summer after phenology data was collected. Branches were cut within a few centimeters of the leaf bud scar that marked the end of the previous year’s growth, which standardized the distance from the tip for the year of the measurement. Because material was limited on the glasshouse grown plants in the salicaceous garden, most of the branches we sampled were 1‐yr‐old and were sampled within a centimeter of their terminal bud. All branches were sectioned with a sliding microtome (10–15 μm). They were stained with 1% safranin O and analyzed using ImageJ (Schneider *et al*., 2012). We measured the area of over 200 vessels in ring porous species (except *Gleditsia triacanthos*, *n* = 141, *Quercus velutina*, *n* = 156, *Ulmus × hollandica*, *n* = 175) and 100 vessels in the remaining species. Average vessel diameter for each sample was calculated assuming the vessels were round in cross‐section. We only measured vessels from the most recent, complete year of growth in each branch because these vessels provide the bulk hydraulic support for the newly developing leaves in the spring (if not embolized). This meant that we sampled the second year of growth in 3‐yr‐old samples and could avoid vessels that expanded after leaf out and protoxylem from the first year. Note that there are species in our study that may have vascular and vasicentric tracheids that cannot be easily differentiated from vessels in cross‐section. We did not characterize these cells separately and grouped them with the vessels. Assuming these conduits are conductive, there should be a similar relationship between their diameter and freezing‐induced embolism as observed in vessels (Davis *et al*., 1999; Pittermann & Sperry, 2003).

We classified each species as diffuse porous (vessels remain a similar width throughout the growth ring), semi‐ring porous (vessels gradually change in size from wider to narrower as the year progresses) or ring porous (wood has two distinct types of vessels: wide earlywood vessels and narrow latewood vessels). Several salicaceous species exhibited differences in wood porosity among samples. We noted when this was the case and for our subsequent analyses considered a species diffuse porous if most samples were diffuse porous.

Previous work has shown that there is a critical vessel diameter (*c*. 30 μm in deciduous angiosperms) over which vessels often exhibit freezing‐induced embolism assuming a water potential of −0.5 MPa (Davis *et al*., 1999). Although this threshold is influenced by water potentials and freezing velocity (Sevanto *et al*., 2012), it serves as a good benchmark for understanding when vessels first become susceptible to freezing‐induced embolism under moderate conditions. Therefore, we directly examined our data in reference to this threshold and also looked at the distribution of vessels within each stem, determining the percent of vessels that were over this critical threshold.

### Dye perfusion

We used a dye perfusion test to examine the amount of active xylem at leaf out in co‐occurring species and examine the relationship between leaf phenology and theoretical hydraulic conductivity. For this analysis, we selected 12 local tree and shrub species from Bagley Nature Area, Duluth, MN, USA and monitored eight individuals per species over a 4‐wk period (7 May–1 June 2018). There was some overlap in species from the anatomy work and the addition of five other local species (Table S4). Between 4 and 8 d after leaf out, one branch from each plant was sampled. Large distal branches (3–5 m) were cut underwater in the morning in the field, bagged and brought back to the laboratory. Branch ends were trimmed and allowed to relax for 2 h while bagged. Prior to taking measurements, we cut a 10 cm stem segment underwater from each stem. These segments were selected to have at least 2 yr of growth (similar to our anatomical sections) and had a diameter of *c*. 5 mm. They were all sampled at least 1 m from the cut end of the branch to minimize potential cutting artifacts. Four stem segments at a time were attached to a manifold filled with water, and dye (1% crystal violet, pH 2) was pulled through the stems following Jacobsen *et al*. (2018). After the dye was observed in tubing on the other side of the stem, stems were flushed and removed from the setup. All samples were processed within 24 h of being collected outside.

We cut each stem in the middle with a razor blade and imaged the entire section with a stereo microscope to determine the cross‐sectional area of wood that contained dye in the current and past growth rings. We also made thin sections (10–15 μm) using a sledge microtome and imaged the samples using a compound microscope (SEBA 2; Laxco, Bothell, WA, USA) to estimate hydraulic conductivity of functional conduits based on Poiseuille’s law (Tyree & Ewers, 1991). For ring and semi‐ring porous species, we estimated theoretical xylem conductivity at 20°C by measuring the area of each vessel that had dye using the equation:

$$K=\;\frac{\pi \rho_{w}}{128\eta }\sum_{i\;=1}^{n}d_{i}^{4}$$

where *ρ*_w_ is the density of water, *η* is the viscosity of water and *d_i_* is the diameter of the *i* ^th^ vessel.

For diffuse porous species, we estimate the average diameter ($\bar{d}$) of a minimum of 100 vessels in each stem along with the vessel density in the region sampled (*D*) and the xylem area that contained dye (*A*). We assumed that vessel width and density were consistent across the sample and then calculated theoretical conductivity of the whole segment using the equation:

$$K=\;\frac{\pi \rho_{w}DA{\bar{d}}^{4}}{128\eta }$$

For one species, *Populus balsamifera*, we did not have high resolution images of four stems. For these stems, we used the average vessel diameter and density of the other three stems in our calculations.

### Species distribution modeling

We modeled the climatic niches of all of the species in our three common gardens based on occurrence data from GBIF.org (Tables S5) and climate data from Worldclim v.2.0, 5 arc‐minutes (Hijmans *et al*., 2005) using the program Maxent, v.3.4.1 (Philips *et al*., 2006). This program estimates the probability that a species will occur in grid‐cells using a maximum entropy model. We ran a 25% test set and our models had a high specificity and a test AUC (area under the curve) greater than 0.86 with the exception of *Salix alaxensis* (AUC = 0.83), *S. fuscescens* (AUC = 0.85) and *Ribes triste* (AUC = 0.82). We found a minimum of 100 collection sites per species with the exception of *Berberis virgetorum* (*n* = 40), *Carya laciniosa* (*n* = 86), *Hemiptelea davidii* (*n* = 76), and *Liriodendron chinense* (*n* = 80). Three plants were not included in this analysis, two were horticultural hybrids (*Ulmus* ‘Patriot’ and *Ulmus* ‘Kansas hybrid’), and one, *Pyrus cossonii* had no georeferenced data points available. We only excluded records that had questionable origin (e.g. appeared outside of the species documented range). We previously ran a similar analysis on willow and poplar species (Savage & Cavender‐Bares, 2013), but we reran the analyses to include data that was not available during the original analysis. We selected the minimum temperature (i.e. variable 6 in the WorldClim data) that had the highest probability of being suitable for the species based on their climatic niche (the zenith of the response curve for this parameter).

### Phylogenetic analysis

We tested whether there was a relationship between leaf phenology (degree days at leaf out) and wood anatomy (average vessel diameter) in our largest dataset (diverse garden) when taking into account species’ phylogenetic relationships. For our phylogeny, we used the most broadly sampled time‐calibrated phylogeny of seed plants to date (Smith & Brown, 2018) to infer the evolutionary relationships of our focal taxa. Specifically, we pruned the most inclusive ALLMB phylogeny with 356 305 taxa to comprise the 64 focal taxa excluding hybrids and cultivars for downstream analyses using the ape v.5.0 package (Paradis & Schliep, 2019) in R v.3.5.1. We chose this approach because attempting to infer the evolutionary relationships of sparsely sampled, nonmonophyletic groups of taxa can lead to spurious results (Park *et al*., 2017, 2018).

We completed a phylogenetic generalized least squares regressions using corPagel correlation structure using the pruned phylogeny with branch lengths described earlier with the ape v.5.4 (Paradis & Schliep, 2019) and nlme v.3 packages (Pinheiro et al., 2021) in R v.3.6.3. We used a base model that estimated *λ* (phylogenetic signal of the residuals) and compared it to a model where *λ* = 0 using *χ* ^2^ likelihood ratio test. This analysis allowed us to test whether the base model was different than a model assuming complete independence (star phylogeny). We also tested for phylogenetic signal in the timing of leaf out and vessel diameter using the ‘phylosig’ function using phytools v.0.7 (Revell, 2012) in R with both Blomberg’s K (Blomberg *et al*., 2003) and Pagel’s *λ* (Pagel, 1999). For each of these tests, we ran 999 randomizations to determine if the model with the calculated signal was different than the one that assumes phylogenetic independence.

### Statistical analyses

In the local and diverse garden, we examined the correlation between three different independent variables (vessel diameter, minimum temperature from the Maxent model and plant height) and growing degree days at the time of leaf out using a least squares multiple regression model. This model was run on a species‐level using average values in the local garden and raw data in the diverse garden where there was no within species replication. Because the variables are likely collinear, we compared all possible linear regression models that involved these variables and selected the model with the lowest Akaike information criterion (AIC). We did a second model that included both datasets and included an interaction parameter (dataset by vessel diameter interaction). For the salicaceous garden analysis, we fit a nonlinear growth model to the relationship between leaf out time and vessel diameter and also completed a logistic regression. We completed similar regression analysis comparing vessel diameter and leaf out time in terms of day of the year. For all our models, we ran them two ways, with raw data and with log‐transformed vessel diameter and height to account for the potential for multiplicative error in these variables (Kerkhoff & Enquist, 2009).

To examine how vessel diameter is influenced by scaling within the plant, we modeled the relationship between vessel diameter, maximum leaf length and plant height using linear regression. Maximum leaf length was determined based on the literature (Petrides, 1972; Takiela, 2001; Smith, 2008; Argus *et al*., 2010; Thomasset *et al*., 2011; Chadde, 2013; Gilmann *et al*., 2018) and data available online through the Missouri Botanical Garden Plant Finder (http://www.missouribotanicalgarden.org/plantfinder/plantfindersearch.aspx, accessed 7 December 2021) and the Flora of China (http://ww.eFloras.org, accessed 7 December 2021). We also examined the relationship between vessel diameter and the minimum temperature in each species climatic niche (based on the Maxent model) directly using regression analysis and by examining the relationship between the residuals of the vessel–leaf length–height relationship and climatic niche. For cross‐species comparison of anatomy and conductivity estimates, we used ANOVA and Tukey honestly significant difference (HSD) multiple comparisons. These analyses were conducted in JMP Pro (v.13.0.0). Maps in this article were created using ArcGIS® software by Esri (Redlands, CA, USA). ArcGIS® and ArcMap™ are the intellectual property of Esri and are used herein under license.

## Results

### Relationship between leaf phenology and vessel diameter

**Fig. 2:**
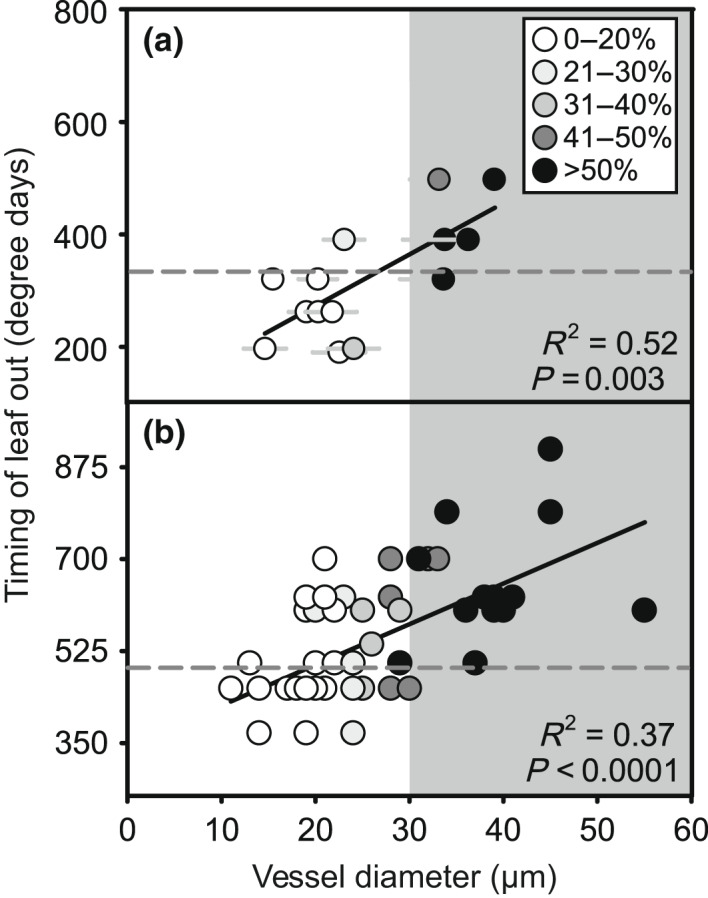
The timing of leaf out correlates with average vessel diameter in woody plants in a local community (a) and a diverse common garden (b). Degree days is the sum of degrees (°C) above 0°C daily starting in January. Each point represents a species average. Symbols are shaded based on the percent of vessels that are over the 30 μm threshold for freezing‐induced embolism. The gray background marks species with an average vessel diameter above the earlier‐mentioned threshold and the dashed line marks the degree days reached at the date when the probability of freezing (temperature of 0°C) was less than 10% at each site.

Time of leaf out in terms of growing degree days was best explained by a model that only included average vessel diameter for the local (*F*_1,12_ = 13.3, *P* = 0.003, *R* ^2^ = 0.52, Fig. 2a) and diverse gardens (*F*_1,53_ = 31.2, *P* < 0.0001, *R* ^2^ = 0.36, Fig. 2b). Model comparisons are in Tables S6–S9. When both datasets were considered in the same model, there was no significant garden by vessel diameter interaction indicating that the best‐fit lines have a similar slope (*F*_2_ = 0.28, *P* = 0.76, Fig. S1). However, the two datasets did have different intercepts and the two species that occur at both sites exhibited earlier leaf out in Duluth, MN, USA compared to Boston, MA, USA in terms of growing degree days (*Acer saccharum* 396 and 454, *Fraxinus pennsylvanica* 326 and 454, respectively). The relationship between leaf out time and vessel diameter was also robust when examining day of the year (diverse garden: *F*_1,53_ = 32.1, *P* < 0.0001, *R* ^2^ = 0.38; local garden: *F*_1,12_ = 12.6, *P* = 0.004, *R* ^2^ = 0.51; Fig. S2). Because temperatures remain cooler later in the year in Duluth, leaf out time in terms of day of the year was earlier in Boston, MA, USA (*Acer saccharum*: 109 and 141, *Fraxinus pennsylvanica*: 109 and 148, respectively). The window for leaf out in these two datasets ranged from 5 to 7 wk with the shortest range occurring in the local garden (6 May–6 June 2019) and the longest in the diverse garden (12 April–24 May 2017). Most of the plants with an average vessel diameter over the critical size threshold for freezing‐induced embolism (30 μm) did not experience leaf out until the risk of freezing was low (< 10% chance of temperatures below 0°C) at each site (Fig. 2).

**Fig. 3:**
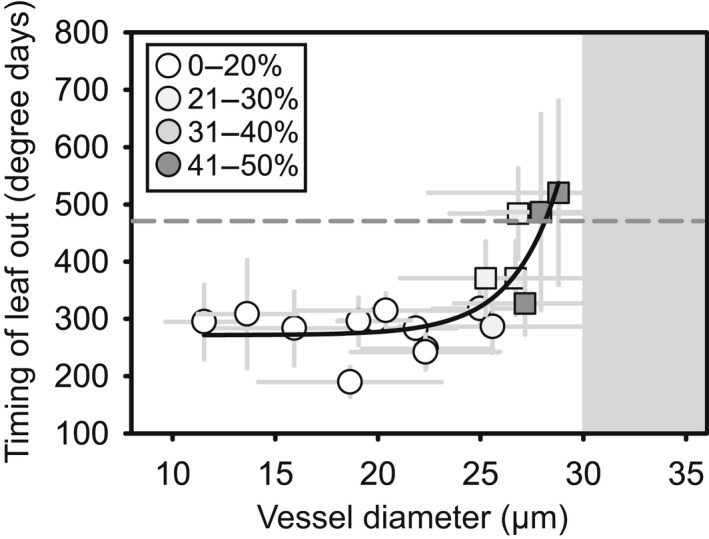
The timing of leaf out exhibits a nonlinear relationship with average vessel diameter in the salicaceous garden. Degree days is the sum of degrees (°C) above 0°C daily starting in January. Each point represents a species average and error bars are ± SD. Squares and circles differentiate species that did and did not exhibit significant dieback in the winter, respectively. Symbols are shaded based on the percent of vessels that are over the 30 μm threshold for freezing‐induced embolism similar to Fig. 2. The gray background marks where the average vessel diameter is above the earlier‐mentioned threshold and the dashed line marks the degree days reached at the date when the probability of freezing (temperature of 0°C) was < 10% at the site. The fit line is an exponential model with the equation $y=y_{0}+a\times e^{\left(b\times x\right)}$).

In contrast to the other datasets, the relationship between growing degree days at leaf out and vessel diameter in the salicaceous garden was nonlinear (logistic regression, *F*_1,12_ = 9.5, *P* = 0.009, *R* ^2^ = 0.44, Fig. 3). In this dataset, the time of leaf out remained fairly constant and only increased for species with an average vessel diameter over 25 μm. Additionally, all of the plants in this garden exhibited leaf out when there was a significant risk of freezing temperatures (> 10% chance of temperatures below 0°C), and during the period we observed leaf out (18 April –2 June 2019), there were 10 d below freezing. As a result, the species with the widest vessels exhibited significant dieback, and a large proportion of these plants only produced new leaves from basal sprouts (Table 1).

**Table 1:** Proportion of plants per species that exhibited leaf out from basal sprouts after winter dieback in the salicaceous garden (*Salix* and *Populus*).

Species^a^	Proportion of individuals with basal sprouts (%)	Mean vessel diameter ± SD (μm)
*Salix caroliniana*	67	27 ± 3
*Salix gooddingii*	50	29 ± 6
*Salix hookeriana*	50	27 ± 5
*Salix sitchensis*	83	27 ± 3
*Populus fremontii*	83	28 ± 3
*Populus balsamifera*	50	25 ± 4

^a^ Only species with dieback are included.

### Phylogenetic analyses

In the diverse garden, the correlation of growing degree days at leaf out with vessel diameter remained significant when phylogenetic relationships were taken into account (*λ* = 0.02, AIC = 619, *t* = 5.4, *P* < 0.00001), and there was no significant difference between the model that included phylogeny and one that assumed complete independence, where *λ* = 0 (*χ* ^2^ = 0.005, *P* = 0.9). We also ran the analysis on local and diverse gardens combined. To combine these datasets, we compared the relationships between leaf out time and vessel diameter. Because the slopes of these regressions were not different among sites, we adjusted the two lines to have the same intercept by adding the difference between the intercepts to the growing degree days of the local species (Fig. S1b). This analysis assumes species exhibit leaf out in the same order across sites and the relationship between leaf out and vessel diameter is not site‐specific. After this adjustment, we reran the phylogenetic generalized least squares using the larger, combined dataset and found that the relationship remained robust (*λ* = 0.01, AIC = 847, *t* = 2.9, *P* = 0.0004) and still was not different than a model assuming phylogenetic independence (*χ* ^2^ = 0.003, *P* = 0.9). We also found little evidence of phylogenetic signal in both average leaf out time (in growing degree days) and vessel diameter using Blomberg’s *K* (*K* = 0.14, *P* = 0.2; *K* = 0.16, *P* = 0.1, respectively) and Pagel’s lambda (*λ* = 0.20, *P* = 0.4; 0.38, *P* = 0.2, respectively) when examining the combined dataset.

### Wood porosity and vessel diameter

**Fig. 4:**
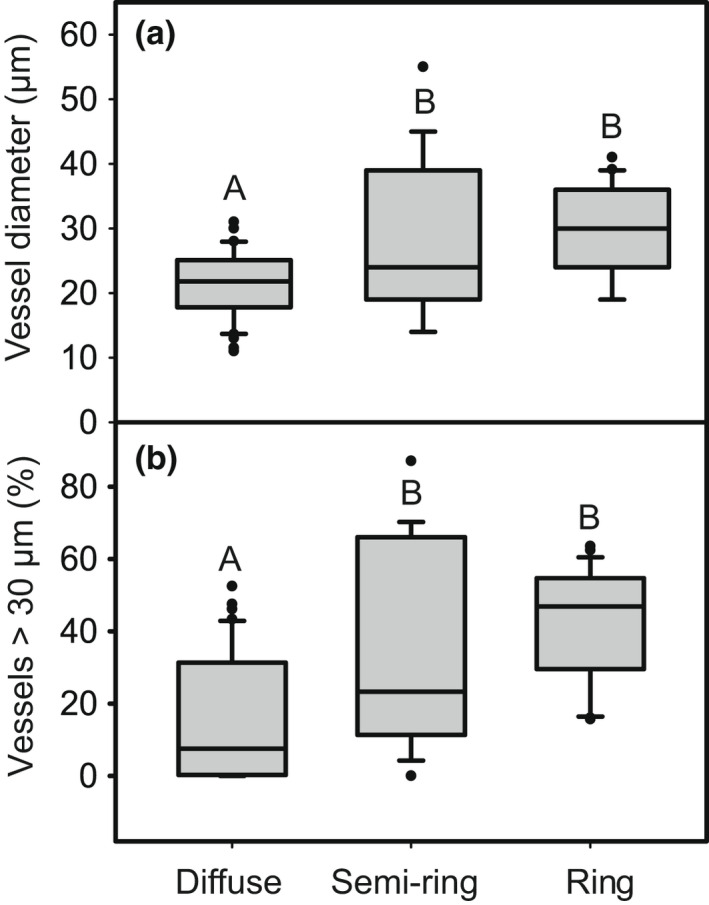
(a) Boxplot of average vessel diameter (μm) and (b) percent of vessels > 30 μm threshold based on woody porosity. Boxes note the 25^th^ and 75^th^ percentile and the median. Bars are the 10^th^ and 90^th^ percentiles, and dots are outliers. Data from all three gardens are included, and the wood porosity was determined based on the samples at each site. Significant differences between gardens was determined using a Tukey HSD (*α* = 0.01) and noted by different letters. The sample sizes for each porosity are: diffuse (*n* = 41), semi‐ring (*n* = 19), and ring (*n* = 27).

There was a significant difference in average vessel diameter based on wood porosity with ring‐porous and semi‐ring porous wood having wider vessels, than diffuse‐porous wood (*F*_2,83_ = 11.9, *P* = 0.0001, Fig. 4a). All but one species (*Magnolia tripetala*) with over 50% of its vessels above the threshold were ring and semi‐ring porous, leading to a significant difference in the width distribution of vessels with wood porosity (*F*_2,83_ = 17, *P* < 0.0001, Fig. 4b). Ring‐porous species tended to leaf out later than diffuse‐porous species on average, but the difference was only significant in the local garden (*F*_2,11_ = 11.7, *P* = 0.002) and not the diverse garden (*F*_2,52_ = 3.0, *P* = 0.06). There were no species with ring‐porous wood in the salicaceous garden. Across all datasets, average vessel diameter linearly correlated with the proportion of vessels > 30 μm (*F*_1,85_ = 706, *P* < 0.0001, *R* ^2^ = 0.89, Fig. S3).

### Seasonal progression of xylem function

**Fig. 5:**
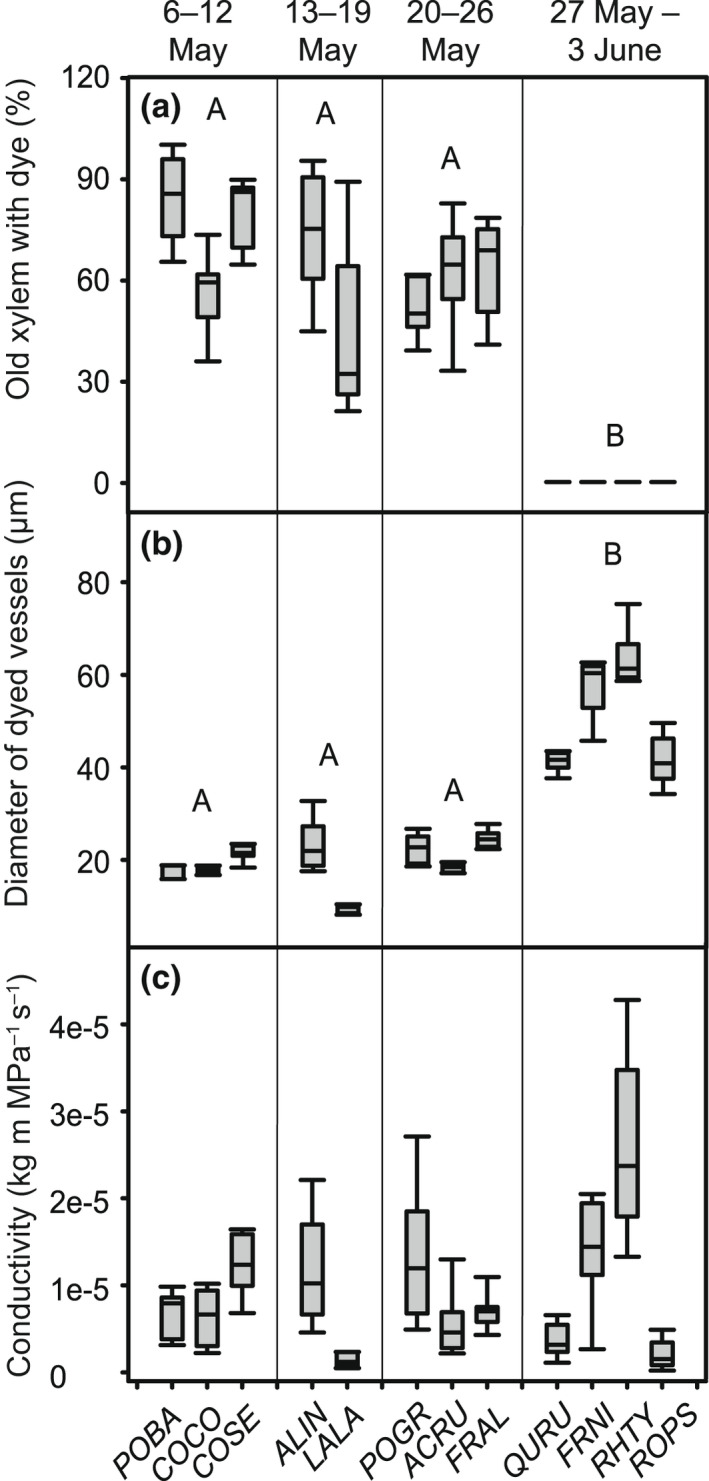
Boxplot of dye perfusion results at leaf out including (a) the percent of xylem cross‐section area from the previous growing season that is functional, i.e. has dye; (b) average diameter of conductive vessels with dye on a species‐level; (c) estimated hydraulic conductivity of stem segments based on the anatomy of the functional vessels with dye. Boxes note the 25^th^ and 75^th^ percentile and the median. Bars are the 10^th^ and 90^th^ percentiles. Species are grouped based on the week of leaf out. Note the last four species to leaf out were sampled the same week as the freeze risk dropped below 10% and were all ring‐porous. Species in order from left to right are *Populus balsamifera*, *Corylus cornuta*, *Cornus sericea*, *Alnus incana*, *Larix laricina*, *Populus grandidentata*, *Acer rubrum*, *Frangula alnus*, *Quercus rubra*, *Fraxinus nigra*, *Rhus typhina* and *Robinia pseudoacacia*. Significant differences among weeks are noted with capital letters (*α* = 0.01).

Dye perfusion tests suggest that the amount of xylem re‐used from previous growing seasons during leaf out is different among species (ANOVA, *F*_3,8_ = 30.2, *P* = 0.0001) and lowest for woody plants that leaf out later in the spring (Fig. 5a). In the four species that experienced leaf out in the last week of monitoring, almost all the dye present in the xylem was observed in newly formed vessels. These species were all ring porous, and as a result, there is a significant difference in dye perfusion based on wood porosity (*t*‐test, *T*_1,10_ = 77, *P* < 0.0001). There was also a significant difference in the diameter of vessels containing dye in the spring (ANOVA, *F*_3,8_ = 14.4, *P* = 0.001) with the latest species to leaf out having dye only in wide, earlywood vessels (Fig. 5b). In these species, the large width of these vessels was able to compensate for the low function of the previous year’s wood, and as a result, there was no significant difference in the hydraulic conductivity of species that exhibited leaf out in different weeks during the spring (ANOVA, *F*_3,8_ = 0.21, *P* = 0.89; Fig. 5c).

### Wood anatomy, plant height and climatic niche

When examining all our data, only leaf length had a significant effect on vessel diameter when both plant height and average maximum leaf length were included in the same model (*F*_1,81_ = 4.64, *P* < 0.0001, Fig. S4a,b). Vessel diameter did not strongly relate to climatic niche (defined by the average minimum temperature in the coldest month, *F*_1,82_ = 4.97, *P* = 0.03, Fig. S4c) but did correlate in the salicaceous garden (*F*_1,16_ = 25.7, *P* = 0.0001). There was no correlation when the residuals of the vessel–leaf length–height relationship was analyzed in relationship to climatic niche (*F*_1,80_ = 4.45, *P* = 0.04).

## Discussion

We found that leaf out time was positively correlated with average vessel diameter across a group of 220 plants regardless of phylogenetic affiliation and species’ geographic origin (Figs 2, 3). We also demonstrated a connection between vessel size, hydraulic conductance, and leaf phenology (Fig. 5) and found that the earliest plants to leaf out had no vessels wider than the threshold for freezing‐induced embolism under moderate water potentials (Figs 2, 3). Taken together, these data provide multiple lines of support for the hydraulic framework proposed by Lechowicz (1984). Although some recent studies have used broad categories to characterize vessel width (Panchen *et al*., 2014; Fahey, 2016), we found that the relationship between vessel diameter and leaf phenology was continuous. However, scatter in the relationship and differences in the fit of this relationship in our three gardens makes it seem unlikely that wood anatomy could serve as an effective proxy for leaf phenology.

### Trade‐offs between freezing tolerance and efficiency could impact phenology

The original framework for understanding the relationship between leaf phenology and wood anatomy was focused on the hydraulic demands of an expanding canopy, but there is another side to the story. If the only restriction on leaf phenology is stem xylem transport capacity, we would find no relationship between vessel diameter and leaf phenology in plants with narrow vessels and limited freezing‐induced embolism, which is in contrast to our findings. In our diverse and local gardens, we observed a linear relationship between leaf out time and vessel diameter, and species with narrow vessels did not display the entire range of leaf out times in each garden (Fig. 2). These results indicate that there is likely a cost to producing leaves later in the season (e.g. lower productivity with shorter growing season) and/or that vessel diameter and leaf phenology are tied to a larger set of trade‐offs between freezing tolerance (i.e. safety) and efficiency (Sperry, 2008; Pratt & Jacobsen, 2017). If this is the case, then plants with wider vessels would have the advantage of a high xylem conductance (per leaf area) but experience greater turnover in functional vasculature (because of embolism) and potentially a shorter growing season than plants with more narrow vessels.

For plants to survive in a seasonally cold habitat, they need to tolerate freezing temperatures, and this requires coordination across different organs (Savage & Cavender‐Bares, 2013). As a result, it is often challenging to causally link one trait related to freezing tolerance (i.e. vessel diameter) to leaf phenology. Therefore, it is possible that the relationship between vessel diameter and leaf phenology is driven by selection for a greater freezing tolerance in plants that leaf out earlier in the spring and not solely by stem vulnerability to freezing‐induced embolism. For example, evidence suggests that leaf phenology may also be linked to leaf freezing tolerance (Lenz *et al*., 2013; Savage & Cavender‐Bares, 2013) and may be different between species with determinant and indeterminant growth. Both of these factors could explain why in other studies some species with small vessels like *Tilia* and *Ulmus* have been found to leaf out later than expected based on their vessel diameter (Lechowicz, 1984).

One place where we saw a clear signal of freezing damage was in the salicaceous garden. We believe the different shape of the relationship between vessel diameter and leaf out time in this garden (Fig. 3) was a result of dieback in wide‐vesseled species caused by late freezing events (Table 1). Dieback can be a result of freezing‐induced embolism in the xylem (Sperry & Pockman, 1993; Cox & Malcolm, 1997), or a result of damage to living tissue (Gonzalez Antivilo *et al*., 2020). Previous work shows that willows are susceptible to winter dieback when planted outside of their range (Sakai, 1970) and their geographic distributions may be limited by their leaf phenology (Savage & Cavender‐Bares, 2013). In our garden, most species at this site produced leaves before the last freeze event, and plants that exhibited dieback before budburst often resprouted from the base of the plant. We believe that it was the resprouting that led to the delay in leaf out in these species.

### Leaf and wood phenology are tied to wood porosity

One factor that could mediate the relationship between wood anatomy and leaf phenology is wood porosity. In general, diffuse‐porous species had earlier leaf out, narrower vessels, and fewer wide vessels compared to ring‐ and semi‐ring‐porous species (Fig. 4). We also found that diffuse‐porous species primarily relied on vessels produced the previous growing season (Fig. 5). This pattern is not surprising considering it is well‐documented that diffuse‐porous species produce leaves before or synchronously with new vessels (Cŭfar *et al*., 2008; Takahashi *et al*., 2015), and ring‐porous species exhibit leaf out after the lignification/maturation of new vessels (Suzuki *et al*., 1996; Kudo *et al*., 2015; Takahashi *et al*., 2015; Kitin & Funada, 2016). As a result, diffuse‐porous species are less likely than ring‐porous species to have new, functional vessels at the time of leaf out.

One proposed explanation for differences between ring‐ and diffuse‐porous species is their sensitivity to auxin, a hormone that appears important in vessel production and expansion (Aloni, 1987; Johnson *et al*., 2018). Some evidence suggests that diffuse‐porous species require higher levels of auxin to initiate xylogenesis than ring‐porous species, causing them to produce vessels only after new leaves become a major auxin source (Aloni *et al*., 1991; Aloni & Peterson, 1997). Meanwhile, ring‐porous species can start xylogenesis while their leaves are still in bud (Aloni *et al*., 1991; Aloni & Peterson, 1997). Therefore, it is possible that differences in auxin responsiveness across species could contribute to some of the unexplained variability in our data but more research is needed to better understand the role of auxin and other hormones in vascular phenology (for review and discussion see Aloni (2015); Hacke *et al*. (2017) and Johnson *et al*. (2018)).

### Implications of sampling design

Sampling design in this type of study is critical because of spatial variation in xylem conduit diameter and vascular phenology along the length of a plant (Kudo *et al*., 2015; M. E. Olson *et al*., 2020). In our study, we focused on the second growth ring of distal branches for two reasons. First, distal branches are expected to show higher embolism than the trunk because trunks are insulated, have a slower velocity of freezing, a less negative water potential and probably experience fewer freeze–thaw events (Mayr *et al*., 2003; Ball *et al*., 2006; Sevanto *et al*., 2012; Charrier *et al*., 2017). Second, by focusing on the second growth ring in branches that were older than 2 yr, we were able to measure the vessels that contributed the most to stem hydraulic conductance at the time of leaf out. We also tried to account for tip‐to‐base widening of vessels by sampling wood at a set distance from the bud scale, which controlled for distance from the tip of the plant at the time of leaf out. We examined the impact that both leaf length and plant height had on vessel diameter and only found leaf length to be important. This is not surprising considering xylem scaling starts at the leaf tip (Lechthaler, 2020). Despite our efforts to control for the complexity of wood anatomy in our design, heterogeneity in the xylem could still be impacting the observed relationship between leaf phenology and wood anatomy because hydraulic conductance in the spring could be impacted by factors not accounted for in this study.

Research has shown that both leaf phenology and wood anatomy can sometimes be more similar among closely related species than among more distantly related ones (Davis *et al*., 2010; Davies *et al*., 2013), and we thus accounted for possible phylogenetic nonindependence in our analyses. However, in our datasets, there was no evidence of phylogenetic signal in leaf out time or vessel diameter. We also found no evidence that phylogenetic relatedness impacted the relationship between leaf out time and vessel diameter. Nonetheless, phenological responses and related traits may be conserved more broadly, as we only examined a small subset of nonrandomly selected taxa and the strength and direction of phylogenetic signal can manifest differently depending on the identity and breadth of species examined. For instance, Park *et al*. (2021) found evidence of phylogenetic signal in flowering time and its sensitivity to temperature overall across all angiosperm taxa in their study, but their results also uncovered large variation in flowering time among closely related species in certain clades. It has also been argued that conflating comparative inferences potentially involving population‐level processes with higher‐level phylogenetic processes can be problematic (Olson, 2021), and in this light, the lack of phylogenetic signal may indicate that the variation in the traits we investigated was generated independently of shared evolutionary history.

### Conclusions

In three common gardens, we show a clear relationship between vessel diameter and leaf out time and found evidence that only plants with vessels below the critical threshold for freezing‐induced embolism produced leaves before the risk of freezing was below 10%. These results are consistent with a larger trade‐off between freezing tolerance and efficiency and support the growing number of studies that indicate selection may limit the width of conduits in cold climates (Gleason *et al*., 2018; M. Olson *et al*., 2020). However, more research is needed to better understand what drives scatter in the fit of this relationship and how it fits into our broader understanding of the connection between leaf–stem hydraulics and plant safety and efficiency trade‐offs.

## Author contributions

JAS, NM and MR designed the experiments. JAS, TK, NM, KM and MR collected data. TK did the species distribution modeling. DP and JAS did phylogenetic analyses. JAS wrote the manuscript with input from all the authors.

## Supporting information


**Fig. S1** Combined analysis of growing degree days and vessel diameter in diverse and local groups.
**Fig. S2** Relationship between leaf phenology (day of year) and vessel diameter.
**Fig. S3** Relationship between percent of vessels over 30 μm and average vessel diameter.
**Fig. S4** Relationship between vessel diameter, height, maximum leaf length and climatic niche.
**Table S1** Data and species list from Bagley Nature Area, Duluth, MN, USA.
**Table S2** Data and species list from the Arnold Arboretum, Boston, MA, USA.
**Table S3** Data and species list from Franklinville, NY, USA.
**Table S4** Data and species list for dye perfusion tests.
**Table S5** Species locations used in modeling climatic niche.
**Table S6** Linear model of growing degree days at leaf out in local group with raw data.
**Table S7** Linear model of growing degree days at leaf out in local group with partially log‐transformed data.
**Table S8** Linear model of growing degree days at leaf out in diverse group with raw data.
**Table S9** Linear model of growing degree days at leaf out in diverse group with partially log‐transformed data.
Please note: Wiley Blackwell are not responsible for the content or functionality of any Supporting Information supplied by the authors. Any queries (other than missing material) should be directed to the *New Phytologist* Central Office.

## Data Availability

The data that support the findings of this study are openly available in the Data Repository for University of Minnesota (DRUM) at https://doi.org/10.13020/sbkp‐kd14.

## References

[nph18041-bib-0001] Aloni R . 1987. Differentiation of vascular tissues. Annual Review of Plant Physiology and Plant Molecular Biology 38: 179–204.

[nph18041-bib-0002] Aloni R . 2015. Ecophysiological implications of vascular differentiation and plant evolution. Trees‐Structure and Function 29: 1–16.

[nph18041-bib-0003] Aloni R , Peterson CA . 1997. Auxin promotes dormancy callose removal from the phloem of Magnolia kobus and callose accumulation and earlywood vessel differentiation in *Quercus robur* . Journal of Plant Research 110: 37–44. 27520042 10.1007/BF02506841

[nph18041-bib-0004] Aloni R , Raviv A , Peterson CA . 1991. The role of auxin in the removal of dormancy callose and resumption of phloem activity in Vitis‐vinifera. Canadian Journal of Botany‐Revue Canadienne de Botanique 69: 1825–1832.

[nph18041-bib-0005] Argus GW , Eckenwalder JE , Kiger RW . 2010. Salicaceae. In: Flora of North America. Magnoliophytas: Salicaceae to Brassicaceae. New York, NY, USA: Oxford University Press, 3–164.

[nph18041-bib-0006] Ball MC , Canny MJ , Huang CX , Egerton JJG , Wolfe J . 2006. Freeze/thaw‐induced embolism depends on nadir temperature: the heterogeneous hydration hypothesis. Plant, Cell & Environment 29: 729–745. 10.1111/j.1365-3040.2005.01426.x 17087458

[nph18041-bib-0007] Blomberg SP , Garland T , Ives AR . 2003. Testing for phylogenetic signal in comparative data: behavioral traits are more labile. Evolution 57: 717–745. 12778543 10.1111/j.0014-3820.2003.tb00285.x

[nph18041-bib-0008] Chadde S . 2013. Minnesota flora: an illustrated guide to the vascular plants of Minnesota. Scotts Valley, CA, USA: CreateSpace Independent Publishing Platform.

[nph18041-bib-0009] Charrier G , Nolf M , Leitinger G , Charra‐Vaskou K , Losso A , Tappeiner U , Ameglio T , Mayr S . 2017. Monitoring of freezing dynamics in trees: a simple phase shift causes complexity. Plant Physiology 173: 2196–2207. 28242655 10.1104/pp.16.01815 PMC5373037

[nph18041-bib-0010] Chave J , Coomes D , Jansen S , Lewis SL , Swenson NG , Zanne AE . 2009. Towards a worldwide wood economics spectrum. Ecology Letters 12: 351–366. 19243406 10.1111/j.1461-0248.2009.01285.x

[nph18041-bib-0011] Choat B , Sack L , Holbrook NM . 2007. Diversity of hydraulic traits in nine *Cordia* species growing in tropical forests with contrasting precipitation. New Phytologist 175: 686–698. 17688584 10.1111/j.1469-8137.2007.02137.x

[nph18041-bib-0012] Cleland EE , Allen JM , Crimmins TM , Dunne JA , Pau S , Travers SE , Zavaleta ES , Wolkovich EM . 2012. Phenological tracking enables positive species responses to climate change. Ecology 93: 1765–1771. 22928404 10.1890/11-1912.1

[nph18041-bib-0013] Cleland EE , Chuine I , Menzel A , Mooney HA , Schwartz MD . 2007. Shifting plant phenology in response to global change. Trends in Ecology & Evolution 22: 357–365. 17478009 10.1016/j.tree.2007.04.003

[nph18041-bib-0014] Cochard H , Coste S , Chanson B , Guehl JM , Nicolini E . 2005. Hydraulic architecture correlates with bud organogenesis and primary shoot growth in beech (*Fagus sylvatica*). Tree Physiology 25: 1545–1552. 16137940 10.1093/treephys/25.12.1545

[nph18041-bib-0015] Cooke JEK , Eriksson ME , Junttila O . 2012. The dynamic nature of bud dormancy in trees: environmental control and molecular mechanisms. Plant, Cell & Environment 35: 1707–1728. 10.1111/j.1365-3040.2012.02552.x 22670814

[nph18041-bib-0016] Cox RM , Malcolm JW . 1997. Effects of duration of a simulated winter thaw on dieback and xylem conductivity of *Betula papyrifera* . Tree Physiology 17: 389–396. 14759847 10.1093/treephys/17.6.389

[nph18041-bib-0017] Cŭfar K , Prislan P , De Luis M , Gričar J . 2008. Tree‐ring variation, wood formation and phenology of beech (*Fagus sylvatica*) from a representative site in Slovenia, SE Central Europe. Trees – Structure and Function 22: 749–758.

[nph18041-bib-0018] Davies TJ , Wolkovich EM , Kraft NJB , Salamin N , Allen JM , Ault TR , Betancourt JL , Bolmgren K , Cleland EE , Cook BI *et al*. 2013. Phylogenetic conservatism in plant phenology. Journal of Ecology 101: 1520–1530.

[nph18041-bib-0019] Davis CC , Willis CG , Primack RB , Miller‐Rushing AJ . 2010. The importance of phylogeny to the study of phenological response to global climate change. Philosophical Transactions of the Royal Society B: Biological Sciences 365: 3201–3213. 10.1098/rstb.2010.0130 PMC2981945 20819813

[nph18041-bib-0020] Davis SD , Sperry JS , Hacke UG . 1999. The relationship between xylem conduit diameter and cavitation caused by freezing. American Journal of Botany 86: 1367–1372. 10523278

[nph18041-bib-0021] Esri . 2021. *Light gray canvas base* [basemap]. Scale not given. *World light gray base*. [WWW document] URL https://www.arcgis.com/home/item.html?id=ed712cb1db3e4bae9e85329040fb9a49 [accessed 17 December 2021].

[nph18041-bib-0022] Fahey RT . 2016. Variation in responsiveness of woody plant leaf out phenology to anomalous spring onset. Ecosphere 7: 1–15.

[nph18041-bib-0023] Flynn D , Wolkovich EM . 2018. Temperature and photoperiod drive spring phenology across all species in a temperate forest community. New Phytologist 219: 1353–1363. 29870050 10.1111/nph.15232

[nph18041-bib-0024] Gilman EF , Watson DG , Klein RW , Koeser AK , Hilbert DR , McLean DC . 2018. Carpinus caroliniana: American hornbeam. Gainesville, FL, USA: IFAS Extension, University of Florida, ENH279.

[nph18041-bib-0025] Gleason SM , Blackman CJ , Gleason ST , McCulloh KA , Ocheltree TW , Westoby M . 2018. Vessel scaling in evergreen angiosperm leaves conforms with Murray’s law and area‐filling assumptions: implications for plant size, leaf size and cold tolerance. New Phytologist 218: 1360–1370. 29603233 10.1111/nph.15116

[nph18041-bib-0026] Gonzalez Antivilo F , Paz RC , Tognetti J , Keller M , Cavagnaro M , Barrio EE , Roig JF . 2020. Winter injury to grapevine secondary phloem and cambium impairs budbreak, cambium activity, and yield formation. Journal of Plant Growth Regulation 39: 1095–1106.

[nph18041-bib-0027] Hacke UG , Sperry JS . 2001. Functional and ecological xylem anatomy. Perspectives in Plant Ecology Evolution and Systematics 4: 97–115.

[nph18041-bib-0028] Hacke UG , Spicer R , Schreiber SG , Plavcová L . 2017. An ecophysiological and developmental perspective on variation in vessel diameter. Plant, Cell & Environment 40: 831–845. 10.1111/pce.12777 27304704

[nph18041-bib-0029] Heide OM . 1993. Dormancy release in beech buds (*Fagus‐sylvatica*) requires both chilling and long days. Physiologia Plantarum 89: 187–191.

[nph18041-bib-0030] Hijmans RJ , Cameron SE , Parra JL , Jones PG , Jarvis A . 2005. Very high resolution interpolated climate surfaces for global land areas. International Journal of Climatology 25: 1965–1978.

[nph18041-bib-0031] Hubbard RM , Ryan MG , Stiller V , Sperry JS . 2001. Stomatal conductance and photosynthesis vary linearly with plant hydraulic conductance in ponderosa pine. Plant, Cell & Environment 24: 113–121.

[nph18041-bib-0032] Hufkens K , Friedl MA , Keenan TF , Sonnentag O , Bailey A , O’Keefe J , Richardson AD . 2012. Ecological impacts of a widespread frost event following early spring leaf‐out. Global Change Biology 18: 2365–2377.

[nph18041-bib-0033] Inouye DW . 2008. Effects of climate change on phenology, frost damage, and floral abundance of montane wildflowers. Ecology 89: 353–362. 18409425 10.1890/06-2128.1

[nph18041-bib-0087] Jacobsen AL , Valdovinos‐Ayala J , Pratt RB . 2018. Functional lifespans of xylem vessels: development, hydraulic function, and post‐function of vessels in several species of woody plants. American Journal of Botany 105: 142–150. 29570215 10.1002/ajb2.1029

[nph18041-bib-0034] Johnson D , Eckart P , Alsamadisi N , Noble H , Martin C , Spicer R . 2018. Polar auxin transport is implicated in vessel differentiation and spatial patterning during secondary growth in *Populus* . American Journal of Botany 105: 186–196. 29578291 10.1002/ajb2.1035

[nph18041-bib-0035] Kerkhoff AJ , Enquist BJ . 2009. Multiplicative by nature: why logarithmic transformation is necessary in allometry. Journal of Theoretical Biology 257: 519–521.

[nph18041-bib-0036] Kitin P , Funada R . 2016. Earlywood vessels in ring‐porous trees become functional for water transport after bud burst and before the maturation of the current – year leaves. IAWA Journal 37: 315–331.

[nph18041-bib-0037] Kudo K , Yasue K , Hosoo Y , Funada R . 2015. Relationship between formation of earlywood vessels and leaf phenology in two ring‐porous hardwoods, *Quercus serrata* and *Robinia pseudoacacia*, in early spring. Journal of Wood Science 61: 455–464.

[nph18041-bib-0038] Lechowicz MJ . 1984. Why do temperate deciduous trees leaf out at different times – adaptation and ecology of forest communities. American Naturalist 124: 821–842.

[nph18041-bib-0039] Lechthaler S , Kiorapostolou N , Pitacco A , Anfodillo T , Petit G . 2020. The total path length hydraulic resistance according to known anatomical patterns: what is the shape of the root‐to‐leaf tension gradient along the plant longitudinal axis? Journal of Theoretical Biology 502: 110369. 32526220 10.1016/j.jtbi.2020.110369

[nph18041-bib-0040] Lenz A , Hoch G , Vitasse Y , Körner C . 2013. European deciduous trees exhibit similar safety margins against damage by spring freeze events along elevational gradients. New Phytologist 200: 1166–1175. 23952607 10.1111/nph.12452

[nph18041-bib-0041] Mayr S , Gruber A , Bauer H . 2003. Repeated freeze–thaw cycles induce embolism in drought stressed conifers (Norway spruce, stone pine). Planta 217: 436–441. 14520570 10.1007/s00425-003-0997-4

[nph18041-bib-0042] Mencuccini M . 2002. Hydraulic constraints in the functional scaling of trees. Tree Physiology 22: 553–565. 12045027 10.1093/treephys/22.8.553

[nph18041-bib-0043] Méndez‐Alonzo RM , Paz H , Zuluaga RC , Rosell JA , Olson ME . 2012. Coordinated evolution of leaf and stem economics in tropical dry forest trees. Ecology 93: 10. 10.1890/11-1213.1 23236911

[nph18041-bib-0044] Menzel A , Fabian P . 1999. Growing season extended in Europe. Nature 397: 659.

[nph18041-bib-0045] Morin X , Lechowicz MJ , Augspurger C , O’Keefe J , Viner D , Chuine I . 2009. Leaf phenology in 22 North American tree species during the 21st century. Global Change Biology 15: 961–975.

[nph18041-bib-0046] Olson ME . 2021. The comparative method is not macroevolution: across‐species evidence for within‐species process. Systematic Biology 70: 1272–1281. 33410880 10.1093/sysbio/syaa086

[nph18041-bib-0047] Olson ME , Anfodillo T , Gleason SM , McCulloh KA . 2020. Tip‐to‐base xylem conduit widening as an adaptation: causes, consequences, and empirical priorities. New Phytologist 229: 1877–1893. 32984967 10.1111/nph.16961

[nph18041-bib-0048] Olson ME , Rosell JA . 2013. Vessel diameter–stem diameter scaling across woody angiosperms and the ecological causes of xylem vessel diameter variation. New Phytologist 197: 1204–1213. 23278439 10.1111/nph.12097

[nph18041-bib-0049] Olson M , Rosell JA , Martínez‐Pérez C , León‐Gómez C , Fajardo A , Isnard S , Cervantes‐Alcayde MA , Echeverría A , Figueroa‐Abundiz VA , Segovia‐Rivas A *et al*. 2020. Xylem vessel‐diameter–shoot‐length scaling: ecological significance of porosity types and other traits. Ecological Monographs 90: e01410.

[nph18041-bib-0050] Osada N . 2017. Relationships between the timing of budburst, plant traits, and distribution of 24 coexisting woody species in a warm‐temperate forest in Japan. American Journal of Botany 104: 550–558. 28424203 10.3732/ajb.1600444

[nph18041-bib-0051] Pagel M . 1999. Inferring the historical patterns of biological evolution. Nature 401: 877–884. 10553904 10.1038/44766

[nph18041-bib-0052] Panchen ZA , Primack RB , Nordt B , Ellwood ER , Stevens AD , Renner SS , Willis CG , Fahey R , Whittemore A , Du YJ *et al*. 2014. Leaf out times of temperate woody plants are related to phylogeny, deciduousness, growth habit and wood anatomy. New Phytologist 203: 1208–1219. 24942252 10.1111/nph.12892

[nph18041-bib-0053] Paradis E , Schliep K . 2019. ape5.0: an environment for modern phylogenetics and evolutionary analyses in R. Bioinformatics 35: 526–528. 30016406 10.1093/bioinformatics/bty633

[nph18041-bib-0054] Park DS , Breckheimer IK , Ellison AM , Lyra GM , Davis CC . 2021. Phenological displacement is uncommon among sympatric angiosperms. New Phytologist 233: 1466–1478. 34626123 10.1111/nph.17784

[nph18041-bib-0055] Park DS , Worthington S , Zhenxiang X . 2017. Taxon sampling and inferred community phylogenies: R replication code and data. Zenodo. doi: 10.5281/ZENODO.1095663.

[nph18041-bib-0056] Park DS , Worthington S , Xi Z . 2018. Taxon sampling effects on the quantification and comparison of community phylogenetic diversity. Molecular Ecology 27: 1296–1308. 29423927 10.1111/mec.14520

[nph18041-bib-0057] Petrides GA . 1972. Trees and shrubs. Boston, NY, USA: Houghton Mifflin Company.

[nph18041-bib-0058] Philips SJ , Anderson RP , Schapire RE . 2006. Maximum entropy modeling of species geographic distributions. Ecological Modelling 190: 231–259.

[nph18041-bib-0059] Pinheiro J , Bates D , DebRoy S , Sarker D ; R Core Team . 2021. nlme: linear and nonlinear mixed effects models. R package v.3.6.3. [WWW document] URL https://CRAN.R‐project.org/package=nlme.

[nph18041-bib-0060] Pittermann J , Sperry J . 2003. Tracheid diameter is the key trait determining the extent of freezing‐induced embolism in conifers. Tree Physiology 23: 907–914. 14532014 10.1093/treephys/23.13.907

[nph18041-bib-0061] Pittermann J , Sperry JS . 2006. Analysis of freeze‐thaw embolism in conifers. The interaction between cavitation pressure and tracheid size. Plant Physiology 140: 374–382. 16377751 10.1104/pp.105.067900 PMC1326058

[nph18041-bib-0062] Polgar CA , Primack RB . 2011. Leaf‐out phenology of temperate woody plants: from trees to ecosystems. New Phytologist 191: 926–941. 21762163 10.1111/j.1469-8137.2011.03803.x

[nph18041-bib-0063] Pratt RB , Jacobsen AL . 2017. Conflicting demands on angiosperm xylem: tradeoffs among storage, transport and biomechanics. Plant, Cell & Environment 40: 897–913. 10.1111/pce.12862 27861981

[nph18041-bib-0064] Prislan P , Gričar J , de Luis M , Smith KT , Cufar K . 2013. Phenological variation in xylem and phloem formation in *Fagus sylvatica* from two contrasting sites. Agricultural and Forest Meteorology 180: 142–151.

[nph18041-bib-0065] Revell LJ . 2012. phytools: an R package for phylogenetic comparative biology (and other things). Methods in Ecology and Evolution 3: 217–223.

[nph18041-bib-0066] Sakai A . 1970. Freezing resistance in willows from different climates. Ecology 51: 485–491.

[nph18041-bib-0067] Saliendra NZ , Sperry JS , Comstock JP . 1995. Influence of leaf water status on stomatal response to humidity, hydraulic conductance, and soil drought in *Betula occidentalis* . Planta 196: 357–366.

[nph18041-bib-0068] Savage JA , Cavender‐Bares JM . 2013. Phenological cues drive an apparent trade‐off between freezing tolerance and growth in the family Salicaceae. Ecology 94: 1708–1717. 24015515 10.1890/12-1779.1

[nph18041-bib-0069] Savage JA , Chuine I . 2021. Coordination of spring vascular and organ phenology in deciduous angiosperms growing in seasonally cold climates. New Phytologist 230: 1700–1715. 33608961 10.1111/nph.17289

[nph18041-bib-0070] Schneider CA , Rasband WS , Eliceiri KW . 2012. NIH image to ImageJ: 25 years of image analysis. Nature Methods 9: 671–675. 22930834 10.1038/nmeth.2089 PMC5554542

[nph18041-bib-0071] Sevanto S , Holbrook NM , Ball M . 2012. Freeze/thaw‐induced embolism: probability of critical bubble formation depends on speed of ice formation. Frontiers in Plant Science 3: 107. 22685446 10.3389/fpls.2012.00107 PMC3368182

[nph18041-bib-0072] Smith SA , Brown JW . 2018. Constructing a broadly inclusive seed plant phylogeny. American Journal of Botany 105: 302–314. 29746720 10.1002/ajb2.1019

[nph18041-bib-0073] Smith W . 2008. Trees and shrubs of Minnesota. Minneapolis, MN, USA: University of Minnesota Press.

[nph18041-bib-0074] Sperry JS , Pockman WT . 1993. Limitation of transpiration by hydraulic conductance and xylem cavitation in *Betula‐Occidentalis* . Plant, Cell & Environment 16: 279–287.

[nph18041-bib-0075] Sperry JS , Sullivan JEM . 1992. Xylem embolism in response to freeze‐thaw cycles and water stress in ring‐porous, diffuse‐porous, and conifer species. Plant Physiology 100: 605–613. 16653035 10.1104/pp.100.2.605 PMC1075601

[nph18041-bib-0076] Sperry . 2008. Safety and efficiency conflicts in hydraulic tecture: scaling from tissues to trees. Plant, Cell & Environment 31: 695. 10.1111/j.1365-3040.2007.01765.x 18088335

[nph18041-bib-0077] Suzuki M , Yoda K , Suzuki H . 1996. Phenological comparison of the onset of vessel formation between ring‐porous and diffuse‐porous deciduous trees in a Japanese temperate forest. IAWA Journal 17: 431–444.

[nph18041-bib-0078] Takahashi S , Okada N , Nobuchi T . 2015. Relationship between vessel porosity and leaf emergence pattern in ring‐ and diffuse‐porous deciduous trees in a temperate hardwood forest. Botany‐Botanique 93: 31–39.

[nph18041-bib-0079] Takiela S . 2001. Trees of Minnesota field guide. Cambridge, MN, USA: Adventure Publications.

[nph18041-bib-0080] Thomasset M , Fernandez‐Manjarrés JF , Douglas GC , Frascaria‐Lacoste N , Raquin C , Hodkinson TR . 2011. Molecular and morphological characterization of reciprocal F1 hybrid and parental species reveals asymmetric character inheritance. International Journal of Plant Sciences 172: 423–433.

[nph18041-bib-0081] Tyree MT , Ewers FW . 1991. The hydraulic architecture of trees and other woody‐plants. New Phytologist 119: 345–360.

[nph18041-bib-0082] Tyree MT , Zimmerman MH . 2002. Xylem structure and the ascent of sap. Germany, Berlin: Springer.

[nph18041-bib-0083] Venturas MD , Sperry JS , Hacke UG . 2017. Plant xylem hydraulics: what we understand, current research, and future challenges. Journal of Integrative Plant Biology 59: 356–389. 28296168 10.1111/jipb.12534

[nph18041-bib-0084] Wang J , Ives NE , Lechowicz MJ . 1992. The relation of foliar phenology to xylem embolism in trees. Functional Ecology 6: 469–475.

[nph18041-bib-0085] Yang S , Tyree MT . 1992. A theoretical model of hydraulic conductivity recovery from embolism with comparison to experimental data on Acer saccharum. Plant, Cell & Environment 15: 633–643.

[nph18041-bib-0086] Yin JJ , Fridley JD , Smith MS , Bauerle TL . 2016. Xylem vessel traits predict the leaf phenology of native and non‐native understorey species of temperate deciduous forests. Functional Ecology 30: 206–214.

