# Supplementary material for: Leaf out time correlates with wood anatomy across large geographic scales and within local communities
Source: New Phytol. 2022 Mar 12;235(3):953–64. doi: 10.1111/nph.18041 (PMC9313884; doi:10.1111/nph.18041)
Supplement: Supplementary file 1 — Fig. S1 Combined analysis of growing degree days and vessel diameter in diverse and local groups. Fig. S2 Relationship between leaf phenology (day of year) and vessel diameter. Fig. S3 Relationship between percent of vessels over 30 μm and average vessel diameter. Fig. S4 Relationship between vessel diameter, height, maximum leaf length and climatic niche. Table S1 Data and species list from Bagley Nature Area, Duluth, MN, USA. Table S2 Data and species list from the Arnold Arboretum, Boston, MA, USA. Table S3 Data and species list from Franklinville, NY, USA. Table S4 Data and species list for dye perfusion tests. Table S5 Species locations used in modeling climatic niche. Table S6 Linear model of growing degree days at leaf out in local group with raw data. Table S7 Linear model of growing degree days at leaf out in local group with partially log‐transformed data. Table S8 Linear model of growing degree days at leaf out in diverse group with raw data. Table S9 Linear model of growing degree days at leaf out in diverse group with partially log‐transformed data. Please note: Wiley Blackwell are not responsible for the content or functionality of any Supporting Information supplied by the authors. Any queries (other than missing material) should be directed to the New Phytologist Central Office. [file NPH-235-953-s001.pdf]

New Phytologist

***New Phytologist* Supporting Information**

Article title: Leaf out time correlates with wood anatomy across large geographic scales and within local communities
Authors: Jessica A. Savage, Thomas Kiecker, Natalie McMann, Daniel Park, Matthew Rothendler, and Kennedy Mosher
Article acceptance date: 24 January 2022

The following Supporting Information is available for this article:

Fig. S1 Combined analysis of growing degree days and vessel diameter in diverse and local groups
Fig. S2 Relationship between leaf phenology (day of year) and vessel diameter
Fig. S3 Relationship between percent of vessels over 30 µm and average vessel diameter
Fig. S4 Relationship between vessel diameter, height, maximum leaf length and climatic niche

Table S1 Data and species list from Bagley Nature Area, Duluth, MN, USA
Table S2 Data and species list from the Arnold Arboretum, Boston, MA, USA
Table S3 Data and species list from Franklinville, NY, USA
Table S4 Data and species list for dye perfusion tests
Table S5 Species locations used in modelling climatic niche
Table S6 Linear model of growing degree days at leaf out in local group with raw data
Table S7 Linear model of growing degree days at leaf out in local group with partially log-transformed data
Table S8 Linear model of growing degree days at leaf out in diverse group with raw data
Table S9 Linear model of growing degree days at leaf out in diverse group with partially log-transformed data

New Phytologist

Fig. S1 . **(a)** The relationship between growing degree days at leaf out and vessel diameter in diverse (black) and local (gray) groups. Each point represents a species average. The slopes for the diverse and local datasets do not show a significant difference, but they do have different intercepts. The best-fit line for each group is shown separately. **(b)** The combined data from both groups when the intercepts are made equal. The best-fit line is shown when all data is included in analysis.

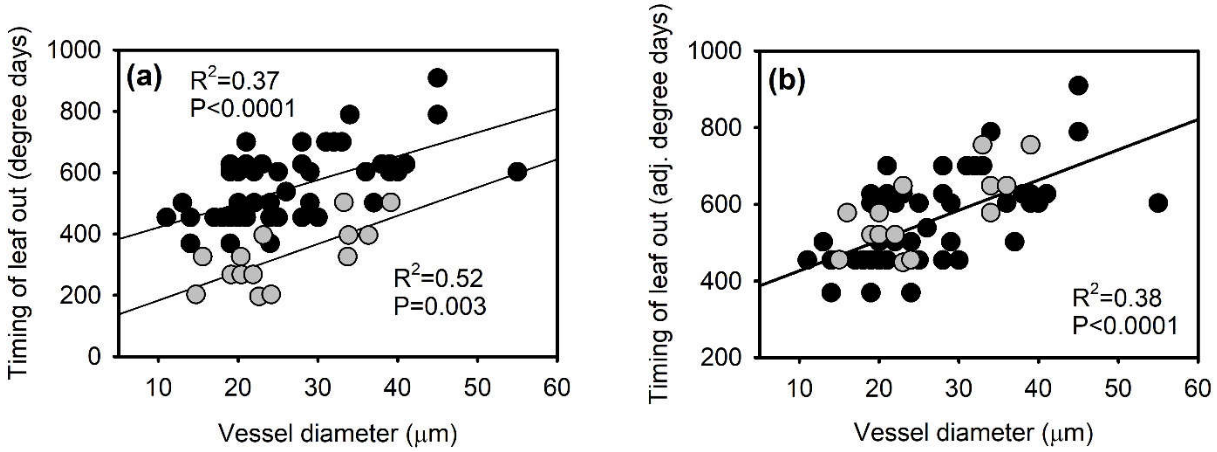

Fig. S2 . The relationship between day of the year at leaf out and vessel diameter in a local group (**a**), a diverse group (**b**) and a closely related group (**c**) of species. Each point represents a species. Data in (a) and (b) are from multiple individuals and graphed as averages with x and y error bars. Symbols are shaded based on the percent of vessels that are over the 30 μm threshold for freezing-induced embolism with darker shades indicating more vessels are vulnerable to embolism. Square symbols note plants that exhibited dieback. The grey background notes wood samples with an average vessel diameter above the threshold and the dashed grey line marks the date when the probability of freezing (temperature of 0°C) was less than 10% at each site.

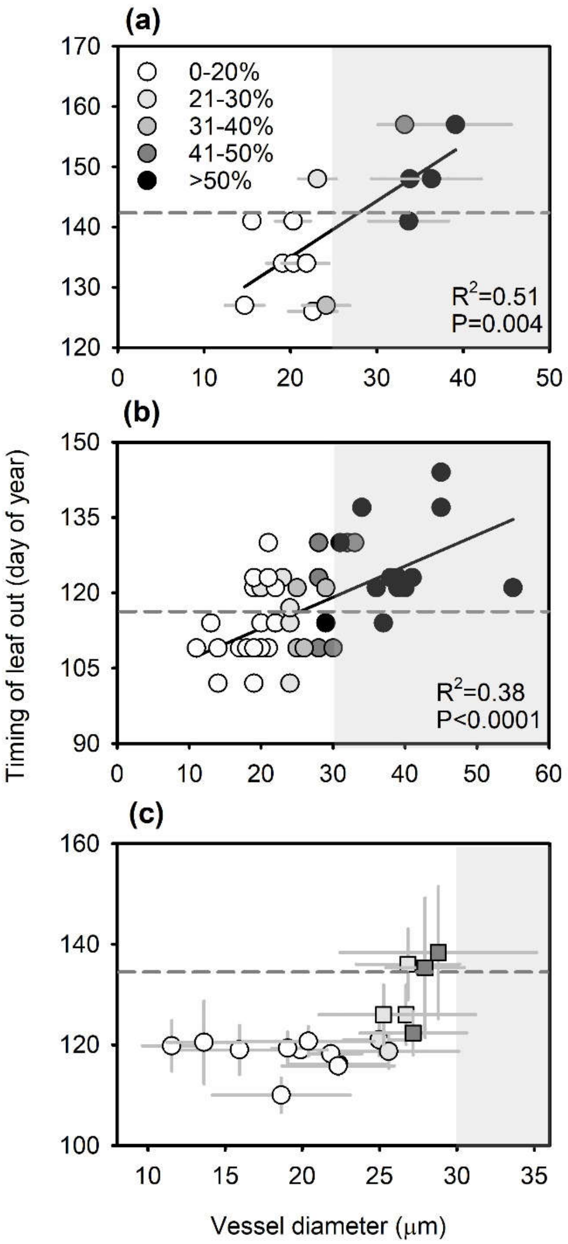

Fig. S3. The relationship between the percent of vessels over 30 µm threshold and average vessel diameter when data from all groups was combined. Each point represents a species average and colors indicate the group: black – diverse, grey – local and white – salicaceous).

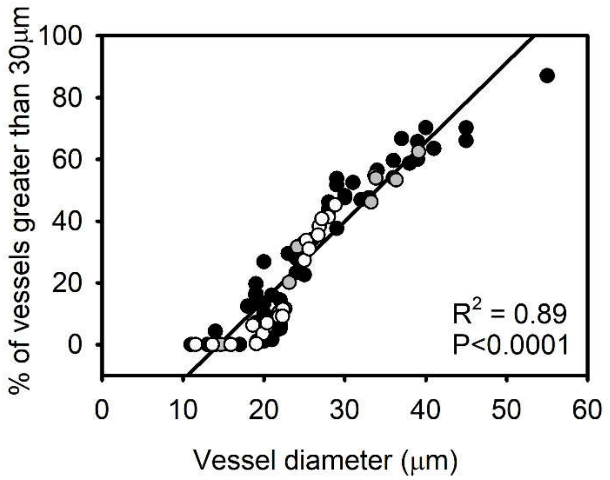

Fig. S4. The relationships between vessel diameter and **(a)** plant height, **(b)** maximum leaf length from the literature, and **(c)** the minimum temperature in species climate niche (Tmin) using data from all three gardens. The only relationship that was significant was between vessel diameter and maximum leaf length. The best-fit line of this relationship is shown on the graph.

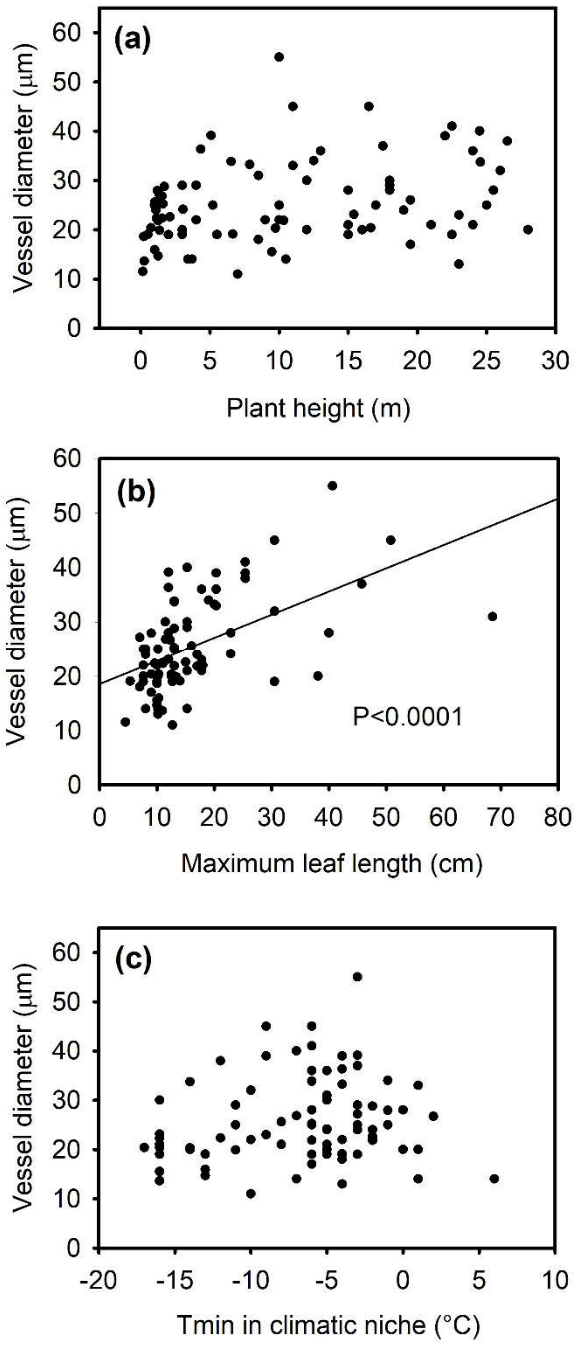

Table S1 Data and species list from Bagley Nature Area, Duluth, MN, USA (local group)*

Species	Min. temp. (°C)	Height (m)	N	Leaf out (day of year)	Leaf out (degree day)	Wood porosity	Vessel D (µm)
Acer saccharum Marshall	-14	10 ± 4	4	141 ± 0	326 ± 0	d	20 ± 2
Alnus incana (L.) Moench *ssp. rugosa* (Du Roi) R.T. Clausen	-4	7 ± 2	4	134 ± 0	268 ± 0	d	19 ± 2
Betula papyrifera Marshall	-16	17 ± 3	4	134 ± 0	268 ± 0	d	20 ± 1
Cornus sericea L. *ssp. sericea*	-2	2 ± 0.5	4	126 ± 0	196 ± 0	d	23 ± 3
Fraxinus nigra Marshall	-14	25 ± 14	4	141 ± 0	326 ± 0	r	34 ± 5
Fraxinus pennsylvanica Marshall	-6	7 ± 6	4	148 ± 0	396 ± 0	r	34 ± 4
Ostrya virginiana (Mill.) K. Koch	-16	9 ± 2	4	141 ± 0	326 ± 0	d	16 ± 1
Parthenocissus vitacea (Knerr) Hitchc.	-3	5 ± 1	4	157 ± 0	503 ± 0	r	39 ± 6
Populus balsamifera L.	-6	10 ± 4	4	134 ± 0	268 ± 0	d	22 ± 3
Populus grandidentata Michx.	-16	15 ± 2	4	148 ± 0	396 ± 0	sr	23 ± 2
Rhus typhina L.	-4	4 ± 1	4	148 ± 0	396 ± 0	r	36 ± 6
Ribes triste Pall.	-13	1.3 ± 0.5	4	127 ± 0	203 ± 0	d	15 ± 2
Robinia pseudoacacia L.	-4	8 ± 5	4	157 ± 0	503 ± 0	r	33 ± 3
Sambucus racemosa L.	-5	3.1 ± 0.2	4	127 ± 0	203 ± 0	d	24 ± 3

* Data is averages ± stdev. Min. temp. is the most probable average minimum temperature in the modeled niche. D is diameter. Wood porosities are ring (r), diffuse (d) or semi-ring porous (sr). Leaf out was from 2019.

Table S2 Data and species list from the Arnold Arboretum, Boston, MA, USA (diverse group)*

Species	Min. temp. (°C)	Accession	Height (m)	Leaf out (day of year)	Leaf out (degree day)	Wood porosity	Vessel D (µm)
Acer saccharum Marshall	-14	20645*A	28	109	454	d	20 ± 7
Aesculus flava Aiton	-5	925-79*B	15	109	454	d	21 ± 6
Albizia julibrissin Durazz.	-6	125-35*A	11	144	909	sr	45 ± 21
Alnus hirsuta (Spach.) Rupr.	-6	1431-77*A	19.5	109	454	d	17 ± 3
Berberis virgetorum C. K. Schneid.	1	341-81-MASS	3.4	102	369	sr	14 ± 6
Betula populifolia Marshall	-10	595-2008*A	10	114	502	d	22 ± 5
Calycanthus floridus L.	-3	1288-73*D	3	114	502	sr	29 ± 8
Caragana arborescens Lam.	-4	912-81*B	2	102	369	sr	19 ± 10
Carpinus caroliniana Walter	-10	1250-79*C	7	109	454	d	11 ± 4
Carya glabra (Mill) Sweet	-10	20095*A	26	130	700	r	32± 16
Carya laciniosa (Michx. f.) G. Don	-9	12898*H	22	121	603	sr	39 ± 18
Carya ovata (Mill.) K. Koch	-9	12907*N	23	123	627	r	23 ± 11
Castanea mollissima Blume	1	7892*A	11	130	700	r	33 ± 19
Catalpa speciosa (Warder) Warder ex. Engelm	-9	1245-79*B	16.5	137	789	sr	45 ± 22
Cephalanthus occidentalis L.	-11	1244-79*MASS-B	4	121	603	r	29 ± 12
Cercidiphyllum japonicum Siebold. & Zucc.	-3	882*A	10	109	454	d	25 ± 6
Cercis canadensis L.	-6	288-93*A	5.5	121	603	r	19 ± 9
Chionanthus retusus Lindl. & Paxton	0	13051*A	12	121	603	r	20 ± 10
Cladrastis kentukea (Dum. Cours.) Rudd	-5	51-87*B	16	114	502	sr	20 ± 7
Cornus macrophylla Wall.	-2	422-94*B	9	121	603	d	22 ± 6
Diospyros virginiana L.	-7	14513*B	24.5	121	603	sr	40 ± 14
Fagus sylvatica L.	-4	17867*A	23	114	502	d	13 ± 4
Fraxinus americana L.	-16	22876*A	22.5	123	627	r	19 ± 111
Fraxinus angustifolia Vahl.	-1	307-58*B	12.5	137	789	r	34 ± 22
Fraxinus pennsylvanica Marshall	-6	617-60*A	25	109	454	r	25 ± 25
Gleditsia triacanthos L.	-5	14681*A	24	121	603	r	36 ± 17
Hemiptelea davidii (Hance) Planch.	-4	14698*A	8.5	109	454	sr	18 ± 9

Ilex decidua Walter	1	151*A	3	109	454	d	20 ± 4
Juglans regia L.	-3	1372-63*A	10	121	603	sr	55 ± 22
Koelreuteria paniculata Laxm.	-3	14928-1*B	17.5	114	502	sr	37 ± 15
Lindera obtusiloba Blume	-4	2602*A	4	114	502	d	22 ± 5
Liriodendron chinense (Hemsl.) Sang.	0	451-80*D	15	130	700	d	28 ± 7
Magnolia tripetala (L.) L.	-5	766-58*A	8.5	130	700	d	31 ± 8
Nyssa sylvatica Marshall	-8	605-2*A	24	130	700	d	21 ± 4
Paeonia suffruticosa Andrews	-2	1300-78*A	1.1	102	369	sr	24 ± 7
Phellodendron amurense Rupr.	-5	143-6*A	12	109	454	r	30 ± 14
Populus deltoides W. Bartram ex. Marshall	-11	22099*A	17	121	603	sr	25 ± 11
Prunus serotina Ehrh.	6	322-2007*A	10.5	109	454	sr	14 ± 5
Pterocarya rhoifolia Siebold & Zucc.	-6	1786-77*B	18	109	454	d	28 ± 13
Pyrus cossonii Rehder	NA	558-85*A	10.5	109	454	d	14 ± 3
Quercus alba L.	-6	631-2008*A	25.5	123	627	r	28 ± 11
Quercus macrocarpa Michx.	-12	16847*M	26.5	123	627	r	37 ± 17
Quercus robur L.	-5	373-96*B	NA	114	502	r	24 ± 14
Quercus rubra L.	-4	22888*A	22	123	627	r	39 ± 20
Quercus velutina Lam.	-6	243-2011*A	22.5	123	627	r	41 ± 20
Sassafras albidum (Nutt.) Nees	-6	22915*A	13	121	603	r	36 ± 19
Symplocos paniculata Miq.	-1	17587-1*D	5.2	109	454	d	25 ± 8
Syringa vulgaris L.	-5	11420*A	3	102	369	sr	19 ± 6
Tilia americana L.	-16	17527*D	18	109	454	d	30 ± 7
Ulmus americana L.	-16	250-53*C	21	123	627	r	21 ± 12
Ulmus ('Patriot') L.	NA	136-98*C	18	121	603	r	29 ± 16
Ulmus ('Kansas Hybrid') L.	NA	362-63*B	19.5	109	538	r	26 ± 14
Ulmus x hollandica Mill.	-3	135-54*B	19	117	454	r	24 ± 10
Viburnum prunifolium L.	-7	1189-85*B	3.7	102	369	d	14 ± 5
Zelkova serrata (Thunb.) Makino	-3	497-79*A	15	109	454	r	19 ± 10

[*] Vessel data is averages within individual plants ± stdev. Min. temp. is the most probable average minimum temperature in the modeled niche. Hybrids were not included. D is diameter. Wood porosities are ring (r), diffuse (d) or semi-ring porous (sr). Note that there is no height data for *Q. robur*, which was cut. Leaf out was from 2017.

Table S3 Data and species list from Franklinville, NY, USA (salicaceous group)*

Species	Origin	Min. temp. (°C)	N	Height (cm)	Leaf out (day of year)	Leaf out (degree day)	Wood porosity	Vessel D(μm)
Salix alaxensis (Andersson) Coville	AK	-33	7	21 ± 8	110 ± 3	190 ± 26	d/sr	19 ± 4
Salix candida Fluegge ex. Willd.	MN	-13	6	100 ± 32	119 ± 5	283 ± 65	d/sr	16 ± 4
Salix caroliniana Michx.	FL	-7	6	152 ± 37	136 ± 7	484 ± 79	d	27 ± 3
Salix eriocephala Michx.	MN	-12	7	153 ± 46	116 ± 2	248 ± 34	sr	22 ± 3
Salix fuscescens Andersson	AK	-36	5	15 ± 5	120 ± 5	294 ± 66	d/sr	12 ± 2
Salix gooddingii C.R. Ball	NV	-2	6	170 ± 21	138 ± 13	521 ± 161	sr	29 ± 6
Salix hookeriana Barratt ex Hook.	OR	2	6	139 ± 41	126 ± 6	371 ± 65	d/sr	27 ± 5
Salix interior Rowlee	MN	-8	7	101 ± 42	119 ± 3	286 ± 46	d	26 ± 5
Salix lasiandra Benth.	OR	-2	5	124 ± 65	118 ± 2	283 ± 31	d	22 ± 2
Salix lucida Muhl.	MN	-11	6	136 ± 41	119 ± 0	297 ± 0	d/sr	20 ± 2
Salix pedicellaris Pursh	MN	-13	6	51 ± 9	119 ± 3	296 ± 42	d	19 ± 1
Salix petiolaris Sm.	MN	-16	5	112 ± 35	116 ± 2	242 ± 31	d/sr	22 ± 4
Salix pseudomyrsinites Andersson	AK	-16	6	25 ± 5	121 ± 38	308 ± 95	d/sr	14 ± 2
Salix pyrifolia Andersson	MN	-17	7	73 ± 31	121 ± 3	315 ± 31	sr	20 ± 4
Salix sitchensis Sanson ex. Bong.	OR	-3	6	133 ± 19	122 ± 4	327 ± 55	d	27 ± 3
Populus balsamifera ssp. trichocarpa Torrey & A. Gray	OR	-6	6	160 ± 50	126 ± 6	371 ± 65	d/sr	25 ± 4
Populus deltoides W. Bartram ex. Marshall	MN	-11	6	98 ± 30	121 ± 3	318 ± 32	d/sr	25 ± 2
Populus fremontii S. Watson	NV	-1	6	120 ± 31	135 ± 14	487 ± 171	d/sr	28 ± 3

[*] Data is averages ± stdev. Origin is source of genotype. Collection information in Savage & Cavender-Bares, 2013. Min. temp. is the most probable average minimum temperature in the modeled niche. D is diameter. Wood porosities are ring (r), diffuse (d) or semi-ring porous (sr). Leaf out was monitored in 2009.

Table S4 Data and species list for dye perfusion tests

Species	Week	N	%old xylem with dye	Diameter dyed vessels (μm)	Conductivity (kg m MPa^{-1} s^{-1})
Acer rubrum L.	3	8	62 ± 15%	18 ± 1	5E-06 ± 4E-06
Alnus incana (L.) Moench *ssp. rugosa* (Du Roi) R.T. Clausen	2	7	73 ± 18%	23 ± 5	1.1E-05 ± 6E-06
Cornus sericea L. *ssp. sericea*	1	7	80 ± 10%	21 ± 2	1.2E-05 ± 4E-06
Corylus cornuta Marshall	1	7	56 ± 12%	17.6 ± 0.7	6E-06 ± 3E-06
Fraxinus nigra Marshall	4	6	0 ± 0%	57 ± 6	1.3E-05 ± 6E-06
Frangula alnus Mill.	3	8	64 ± 14%	24 ± 2	7E-06 ± 2E-06
Larix laricina (Du Roi) K. Koch	2	5	43 ± 27%	9.4 ± 0.9	1.5E-06 ± 8E-07
Populus balsamifera L.	1	6	84 ± 13%	18 ± 1*	7E-06 ± 3E-06
Populus grandidentata Michx.	3	6	52 ± 8%	22 ± 3	1.3E-05 ± 8E-06
Quercus rubra L.	4	6	0 ± 0%	41 ± 2	4E-06 ± 2E-06
Rhus typhina L.	4	6	0 ± 0%	63 ± 6	3E-05 ± 1E-05
Robinia pseudoacacia L.	4	6	0 ± 0%	41 ± 6	2E-06 ± 1E-06

* For vessel diameter measurements N=3. Data is averages ± stdev.

Table S5 Occurrence data DOI and date of download from the Global Biodiversity Information Facility (GBIF) * along with estimates of leaf length from the literature.

Species	Date	DOI	Maximum leaf length (cm)	Literature source
Acer saccharum Marshall	19-Apr-20	10.15468/dl.txxa4g	12.5	(Takiela, 2001)
Aesculus flava Aiton	7-Feb-19	10.15468/dl.eeauux	17.78	(Missouri Botanical Garden Plant Finder, 2021)
Albizia julibrissin Durazz.	28-Feb-19	10.15468/dl.9ekkkx	50.8	(Missouri Botanical Garden Plant Finder, 2021)
Alnus hirsuta (Spach.)Rupr.	7-Feb-19	10.15468/dl.nntbxt	9	(Flora of China, 2021)
Alnus incana (L.) Moench *ssp. rugosa* (Du Roi) R.T. Clausen	23-May-19	10.15468/dl.1etxt6	14	(Chadde, 2013)
Berberis virgetorum C. K. Schneider	7-Mar-19	10.15468/dl.7pygrd	8	(Flora of China, 2021)
Betula papyrifera Marshall	23-May-19	10.15468/dl.7alp1a	9	(Chadde, 2013)
Betula populifolia Marshall	7-Feb-19	10.15468/dl.8zv9ds	7.62	(Missouri Botanical Garden Plant Finder, 2021)
Calycanthus floridus K.	8-Feb-19	10.15468/dl.dasuu3	15.24	(Missouri Botanical Garden Plant Finder, 2021)
Caragana arborescens Lam.	28-Feb-19	10.15468/dl.ah5zpm	7.62	(Missouri Botanical Garden Plant Finder, 2021)
Carpinus caroliniana Walter	7-Feb-19	10.15468/dl.mtjfky	12.7	(Gilman *et al.*, 2018)
Carya glabra P. Mill	26-Feb-19	10.15468/dl.dw69vm	30.48	(Missouri Botanical Garden Plant Finder, 2021)
Carya laciniosa (Michx. f.) G. Don	8-Feb-19	10.15468/dl.ujw8ol	20.32	(Missouri Botanical Garden Plant Finder, 2021)
Carya ovata (Mill.) K. Koch	25-Feb-19	10.15468/dl.y3nrsn	17.78	(Missouri Botanical Garden Plant Finder, 2021)
Castanea mollissima Blume	11-Feb-19	10.15468/dl.1skttu	20.32	(Missouri Botanical Garden Plant Finder, 2021)
Catalpa speciosa (Warder) Warder ex. Engelm	11-Feb-19	10.15468/dl.ol42rj	30.48	(Missouri Botanical Garden Plant Finder, 2021)
Cephalanthus occidentalis L.	28-Feb-19	10.15468/dl.us0xwy	15.24	(Missouri Botanical Garden Plant Finder, 2021)
Cercidiphyllum japonicum Siebold. & Zucc.	18-Feb-19	10.15468/dl.trqrkg	10.16	(Missouri Botanical Garden Plant Finder, 2021)
Cercis canadensis L.	18-Feb-19	10.15468/dl.4ec8w7	12.7	(Missouri Botanical Garden Plant Finder, 2021)
Chionanthus retusus Lindl. & Paxton	18-Feb-19	10.15468/dl.3ycnug	10.16	(Missouri Botanical Garden Plant Finder, 2021)
Cladrastis kentukea (Dum. Cours.) Rudd	18-Feb-19	10.15468/dl.s6upnv	38.1	(Missouri Botanical Garden Plant Finder, 2021)
Cornus macrophylla Wall.	18-Feb-19	10.15468/dl.nugmfq	18	(Flora of China, 2021)
Cornus sericea L. *ssp. sericea*	23-May-19	10.15468/dl.7wpn1q	15	(Chadde, 2013)
Diospyros virginiana L.	4-Mar-19	10.15468/dl.zlqtqt	15.24	(Missouri Botanical Garden Plant Finder, 2021)
Fagus sylvatica L.	4-Mar-19	10.15468/dl.q6p6ip	10.16	(Missouri Botanical Garden Plant Finder, 2021)
Fraxinus americana L.	25-Feb-19	10.15468/dl.acdhu2	30.48	(Petrides, 1972)
Fraxinus angustifolia Vahl.	18-Feb-19	10.15468/dl.5xzr1c	19	(Thomasset *et al.*, 2011)
Fraxinus nigra Marshall	28-May-19	10.15468/dl.q5wggu	13	(Chadde, 2013)

Fraxinus pennsylvanica Marshall	28-May-19	10.15468/dl.vz6yqf	13	(Chadde, 2013)
Gleditsia triacanthos L.	19-Feb-19	10.15468/dl.kw5141	20.32	(Missouri Botanical Garden Plant Finder, 2021)
Hemiptelea davidii (Hance) Planch.	4-Mar-19	10.15468/dl.yimrvo	7	(Flora of China, 2021)
Ilex decidua Walter	19-Feb-19	10.15468/dl.f9vtas	7.62	(Missouri Botanical Garden Plant Finder, 2021)
Juglans regia L.	19-Feb-19	10.15468/dl.mlyuyv	40.64	(Missouri Botanical Garden Plant Finder, 2021)
Koelreuteria paniculata Laxm.	4-Mar-19	10.15468/dl.euehuw	45.72	(Missouri Botanical Garden Plant Finder, 2021)
Lindera obtusiloba Blume.	19-Feb-19	10.15468/dl.txl6lr	10	(Flora of China, 2021)
Liriodendron chinense (Hemli.) Sang.	3-Mar-20	10.15468/dl.szspwy	12	(Flora of China, 2021)
Magnolia tripetala (L.) L.	19-Feb-19	10.15468/dl.rmcupt	68.58	(Petrides, 1972)
Nyssa sylvatica Marshall	19-Feb-19	10.15468/dl.bfd318	15.24	(Petrides, 1972)
Ostrya virginiana (Mill.) K. Koch	19-Apr-20	10.15468/dl.v9mw2z	10	(Takiela, 2001)
Paeonia suffruticosa Andrews	3-Mar-20	10.15468/dl.ufhfg6	8	(Flora of China, 2021)
Parthenocissus vitacea (Knerr) Hitchc.	19-Apr-20	10.15468/dl.mke8cj	12	(Smith, 2008)
Phellodendron amurense Rupr.	19-Feb-19	10.15468/dl.zzmvbe	11.43	(Missouri Botanical Garden Plant Finder, 2021)
Populus balsamifera L.	23-May-19	10.15468/dl.qxcnri	13	(Chadde, 2013)
Populus balsamifera ssp. trichocarpa Torrey & A. Gray	7-Mar-19	10.15468/dl.kdpcpp	13	(Chadde, 2013)
Populus deltoides W. Bartram ex. Marshall	19-Feb-19	10.15468/dl.6yqnwh	8	(*Magnoliophytas: Salicaceae to Brassicaceae*, 2010)
Populus fremontii S. Watson	7-Mar-19	10.15468/dl.kdpcpp	9	(*Magnoliophytas: Salicaceae to Brassicaceae*, 2010)
Populus grandidentata Michx.	28-May-19	10.15468/dl.22fwuh	12	(Chadde, 2013)
Prunus serotina Ehrn.	19-Feb-19	10.15468/dl.xyc7bm	15.24	(Petrides, 1972)
Pterocarya rhoifolia Siebold. & Zucc.	5-Mar-19	10.15468/dl.0hw7sm	40	(Flora of China, 2021)
Quercus alba L.	26-Feb-19	10.15468/dl.vo7uwa	22.86	(Petrides, 1972)
Quercus macrocarpa Michx.	5-Mar-19	10.15468/dl.0i2uig	25.4	(Petrides, 1972)
Quercus robur L.	5-Mar-19	10.15468/dl.siyfcv	17	(Flora of China, 2021)
Quercus rubra L.	5-Mar-19	10.15468/dl.xn3hzz	25.4	(Petrides, 1972)
Quercus velutina Lam.	19-Feb-19	10.15468/dl.zaa4u7	25.4	(Petrides, 1972)
Rhus typhina L.	28-May-19	10.15468/dl.6mnjus	12	(Chadde, 2013)
Ribes triste Pall.	20-Apr-20	10.15468/dl.qyutrj	10	(Chadde, 2013)
Robinia pseudoacacia L.	28-May-19	10.15468/dl.nwdvpj	20	(Smith, 2008)
Salix alaxensis (Andersson) Coville	7-Mar-19	10.15468/dl.uogx1x	10	(*Magnoliophytas: Salicaceae to Brassicaceae*, 2010)
Salix candida Fluegge ex. Willd.	7-Mar-19	10.15468/dl.4pgtz5	10.3	(*Magnoliophytas: Salicaceae to Brassicaceae*, 2010)
Salix caroliniana Michx.	7-Mar-19	10.15468/dl.ofdsdr	11.5	(*Magnoliophytas: Salicaceae to Brassicaceae*, 2010)
Salix eriocephala Michx.	7-Mar-19	10.15468/dl.lf19ie	9.6	(*Magnoliophytas: Salicaceae to Brassicaceae*, 2010)

Salix fuscescens Andersson	7-Mar-19	10.15468/dl.mbwhgm	4.5	(*Magnoliophytas: Salicaceae to Brassicaceae*, 2010)
Salix gooddingii C.R. Ball	18-Mar-19	10.15468/dl.swbrok	13	(*Magnoliophytas: Salicaceae to Brassicaceae*, 2010)
Salix hookeriana Barratt ex Hook.	18-Mar-19	10.15468/dl.xwvzaq	12.3	(*Magnoliophytas: Salicaceae to Brassicaceae*, 2010)
Salix interior Rowlee	18-Mar-19	10.15468/dl.rwzqt9	16	(*Magnoliophytas: Salicaceae to Brassicaceae*, 2010)
Salix lasiandra Benth.	18-Mar-19	10.15468/dl.kv9ybu	17	(*Magnoliophytas: Salicaceae to Brassicaceae*, 2010)
Salix lucida Muhl.	18-Mar-19	10.15468/dl.ixgwon	13.3	(*Magnoliophytas: Salicaceae to Brassicaceae*, 2010)
Salix pedicellaris Pursh	21-Mar-19	10.15468/dl.rtqojo	5.3	(*Magnoliophytas: Salicaceae to Brassicaceae*, 2010)
Salix petiolaris Sm.	21-Mar-19	10.15468/dl.olyonx	11	(*Magnoliophytas: Salicaceae to Brassicaceae*, 2010)
Salix pseudomyrsinites Andersson	21-Mar-19	10.15468/dl.xgryuj	10.9	(*Magnoliophytas: Salicaceae to Brassicaceae*, 2010)
Salix pyrifolia Andersson	21-Mar-19	10.15468/dl.dbc3lc	10.3	(*Magnoliophytas: Salicaceae to Brassicaceae*, 2010)
Salix sitchensis Sanson ex. Bong.	21-Mar-19	10.15468/dl.xradge	7	(*Magnoliophytas: Salicaceae to Brassicaceae*, 2010)
Sambucus racemosa L.	5-Jul-19	10.15468/dl.7ajmz6	22.86	(Missouri Botanical Garden Plant Finder, 2021)
Sassafras albidum (Nutt.) Nees	21-Feb-19	10.15468/dl.7q8ot2	17.78	(Missouri Botanical Garden Plant Finder, 2021)
Symplocos paniculata Miq.	5-Mar-19	10.15468/dl.9rbtn4	7.62	(Missouri Botanical Garden Plant Finder, 2021)
Syringa vulgaris L.	19-Feb-19	10.15468/dl.q8uzkv	10	(Chadde, 2013)
Tilia americana L.	25-Feb-19	10.15468/dl.0yixkd	15.24	(Missouri Botanical Garden Plant Finder, 2021)
Ulmus americana L.	5-Mar-19	10.15468/dl.bgdmyq	15.24	(Petrides, 1972)
Ulmus x hollandica Mill.	25-Feb-19	10.15468/dl.o1ouvg	NA	NA
Viburnum prunifolium L.	21-Feb-19	10.15468/dl.oxwyhq	10.16	(Missouri Botanical Garden Plant Finder, 2021)
Zelkova serrata (Thunb.) Makino	25-Feb-19	10.15468/dl.0ze1x0	10	(Flora of China, 2021)

* GBIF data was used for modelling species' climatic niches with Maxent. Species that had no occurrences listed in GBIF (i.e., *Pyrus cossonii*) and cultivars (i.e., *Ulmus* 'Patriot' and *Ulmus* 'Kansas Hybrid') were excluded from this analysis and noted as NA.

Citations:

Argus GW, Eckenwalder JE, Kiger RW. 2010. Salicaceae. In: *Flora of North America. Magnoliophytas: Salicaceae to Brassicaceae*. New York, NY: Oxford University Press, 3–164.

Chadde S. 2013. *Minnesota flora: An illustrated guide to the vascular plants of Minnesota*. USA: CreateSpace Independent Publishing Platform.

Flora of Chine, http://ww.eFloras.org, accessed 7 Dec. 2021

Missouri Botanical Garden Plant Finder, http://www.missouribotanicalgarden.org/plantfinder/plantfindersearch.aspx, accessed 7 Dec. 2021.

Petrides GA. 1972. *Trees and shrubs*. Boston, NY, USA: Houghton Mifflin Company.

Smith W. 2008. *Trees and shrubs of Minnesota*. Minneapolis, MN, USA: University of Minnesota Press.

Takiela S. 2001. *Trees of Minnesota field guide*. Cambridge, MN, USA: Adventure Publications.

Thomasset M, Fernandez-Manjarrés JF, Douglas GC, Frascaria-Lacoste N, Raquin C, Hodkinson TR. 2011. Molecular and morphological characterization of reciprocal F1 hybrid and parental species reveals asymmetric character inheritance. *International Journal of Plant Sciences* **172**: 423–433.

Gilman EF, Watson DG, Klein RW, Koeser AK, Hilbert DR, McLean DC. 2018. *Carpinus cacroliniana: American hornbeam.* Gainesville, FL, USA: IFAS Extension, University of Florida. ENH279.

Table S6 Linear model of growing degree days at leaf out in local group with raw data

Parameter	R^2	RMSE	AIC
Vessel diameter*	0.52	73	166
Vessel diameter & climatic niche	0.58	71.6	168
Vessel diameter & plant height	0.53	75.6	170
Vessel diameter, plant height & climatic niche	0.6	73.4	173
Plant height & climatic niche	0.11	105	179
Plant height	0.02	105	176
Climate niche	0.02	105	176

* Indicates the model with the lowest AIC

Table S7 Linear model of growing degree days at leaf out in local group with partially log-transformed data

Parameter	R^2	RMSE	AIC
Log(Vessel diameter)*	0.48	76.3	167
Log(Vessel diameter) & log(plant height)	0.53	75.7	170
Log(Vessel diameter), log(plant height) & climatic niche	0.54	78.4	170
Log(Vessel diameter) & climatic niche	0.53	75.5	170
Log(Plant height)	0.12	99.2	175
Log (Plant height) & climatic niche	0.26	95.6	176
Climate niche	0.02	105	176

* Indicates the model with the lowest AIC

Table S8 Linear model of growing degree days at leaf out in diverse group with raw data

Parameter	R^2	RMSE	AIC
Vessel diameter*	0.36	98.3	617
Vessel diameter & plant height	0.4	96.3	623
Vessel diameter, plant height & climatic niche	0.4	97.1	619
Vessel diameter & climatic niche	0.38	98.2	618
Plant height	0.12	115	633
Plant height & climatic niche	0.12	116	636
Climate niche	0.03	121	638

* Indicates the model with the lowest AIC

Table S9 Linear model of growing degree days at leaf out in diverse group with partially log-transformed data

Parameter	R^2	RMSE	AIC
Log(Vessel diameter)*	0.36	98.3	617
Log(Vessel diameter) & log(plant height)	0.43	93.7	614
Log(Vessel diameter), log(plant height) & climatic niche	0.43	94.7	616
Log(Vessel diameter) & climatic niche	0.38	98.5	619
Log(Plant height)	0.19	111	630
Log (Plant height) & climatic niche	0.19	111	632
Climate niche	0.03	121	638

* Indicates the model with the lowest AIC
